# Large-scale transcriptome-wide association study identifies new prostate cancer risk regions

**DOI:** 10.1038/s41467-018-06302-1

**Published:** 2018-10-04

**Authors:** Nicholas Mancuso, Simon Gayther, Alexander Gusev, Wei Zheng, Kathryn L. Penney, Zsofia Kote-Jarai, Rosalind Eeles, Matthew Freedman, Christopher Haiman, Bogdan Pasaniuc, The PRACTICAL consortium, Brian E. Henderson, Sara Benlloch, Fredrick R. Schumacher, Ali Amin Al Olama, Kenneth Muir, Sonja I. Berndt, David V. Conti, Fredrik Wiklund, Stephen Chanock, Victoria L. Stevens, Catherine M. Tangen, Jyotsna Batra, Judith Clements, Henrik Gronberg, Nora Pashayan, Johanna Schleutker, Demetrius Albanes, Stephanie Weinstein, Alicja Wolk, Catharine West, Lorelei Mucci, Géraldine Cancel-Tassin, Stella Koutros, Karina Dalsgaard Sorensen, Lovise Maehle, David E. Neal, Freddie C. Hamdy, Jenny L. Donovan, Ruth C. Travis, Robert J. Hamilton, Sue Ann Ingles, Barry Rosenstein, Yong-Jie Lu, Graham G. Giles, Adam S. Kibel, Ana Vega, Manolis Kogevinas, Jong Y. Park, Janet L. Stanford, Cezary Cybulski, Børge G. Nordestgaard, Hermann Brenner, Christiane Maier, Jeri Kim, Esther M. John, Manuel R. Teixeira, Susan L. Neuhausen, Kim De Ruyck, Azad Razack, Lisa F. Newcomb, Davor Lessel, Radka Kaneva, Nawaid Usmani, Frank Claessens, Paul A. Townsend, Manuela Gago-Dominguez, Monique J. Roobol, Florence Menegaux, Kay-Tee Khaw, Lisa Cannon-Albright, Hardev Pandha, Stephen N. Thibodeau, David J. Hunter, Peter Kraft

**Affiliations:** 1 Department of Pathology and Laboratory Medicine, David Geffen School of Medicine, University of California, Los Angeles, Los Angeles, 90095 CA USA; 2 The Center for Bioinformatics and Functional Genomics, Cedars-Sinai Medical Center, Los Angeles, 90048 CA USA; 3 Dana Farber Cancer Institute, Boston, 02215 MA USA; 4 Division of Epidemiology, Department of Medicine, Vanderbilt Epidemiology Center, Vanderbilt-Ingram Cancer Center, Vanderbilt University School of Medicine, Nashville, 37232 TN USA; 5 Department of Epidemiology, Harvard T.H. Chan School of Public Health, Boston, 02115 MA USA; 6 Channing Division of Network Medicine, Department of Medicine, Brigham and Women’s Hospital/Harvard Medical School, Boston, 02115 MA USA; 7 Division of Genetics and Epidemiology, The Institute of Cancer Research, London, SW7 3RP UK; 8 Royal Marsden NHS Foundation Trust, London, SW3 6JJ UK; 9 Department of Medical Oncology, Dana-Farber Cancer Institute and Harvard Medical School, Boston, 02215 MA USA; 10 Department of Preventive Medicine, Norris Comprehensive Cancer Center, Keck School of Medicine, University of Southern California, Los Angeles, 90015 CA USA; 11 Department of Human Genetics, David Geffen School of Medicine, University of California, Los Angeles, Los Angeles, 90095 CA USA; 12 Bioinformatics Interdepartmental Program, University of California, Los Angeles, Los Angeles, 90095 CA USA; 13 Centre for Cancer Genetic Epidemiology, Department of Public Health and Primary Care, University of Cambridge, Strangeways Research Laboratory, Cambridge, CB1 8RN UK; 14 Department of Epidemiology and Biostatistics, Case Western Reserve University, Cleveland, 44106-7219 OH USA; 15 Seidman Cancer Center, University Hospitals, Cleveland, 44106 OH USA; 16 University of Cambridge, Department of Clinical Neurosciences, Cambridge, CB2 0QQ UK; 17 Institute of Population Health, University of Manchester, Manchester, M13 9PL UK; 18 Warwick Medical School, University of Warwick, Coventry, CV4 7AL UK; 19 Division of Cancer Epidemiology and Genetics, National Cancer Institute, NIH, Bethesda, 21701 MD USA; 20 Department of Medical Epidemiology and Biostatistics, Karolinska Institute, Stockholm, SE-171 77 Sweden; 21 Epidemiology Research Program, American Cancer Society, 250 Williams Street, Atlanta, 30303 GA USA; 22 SWOG Statistical Center, Fred Hutchinson Cancer Research Center, Seattle, 98109-1024 WA USA; 23 Australian Prostate Cancer Research Centre-Qld, Institute of Health and Biomedical Innovation and School of Biomedical Science, Queensland University of Technology, Brisbane, 4059 Queensland Australia; 24 Translational Research Institute, Brisbane, 4102 Queensland Australia; 25 University College London, Department of Applied Health Research, London, WC1E 7HB UK; 26 Centre for Cancer Genetic Epidemiology, Department of Oncology, University of Cambridge, Strangeways Laboratory, Cambridge, WC1E 7HB UK; 27 Department of Medical Biochemistry and Genetics, Institute of Biomedicine, University of Turku, Turku, FI-20014 Finland; 28 Tyks Microbiology and Genetics, Department of Medical Genetics, Turku University Hospital, Hospital, 20521 Finland; 29 BioMediTech, University of Tampere, Tampere, FI-33014 Finland; 30 Division of Nutritional Epidemiology, Institute of Environmental Medicine, Karolinska Institutet, SE-171 77 Sweden; 31 Institute of Cancer Sciences, University of Manchester, Manchester Academic Health Science Centre, Radiotherapy Related Research, The Christie Hospital NHS Foundation Trust, Manchester, M13 9PL UK; 32 CeRePP, Pitie-Salpetriere Hospital, Paris, F-75020 France; 33 UPMC Univ Paris 06, GRC N°5 ONCOTYPE-URO, CeRePP, Tenon Hospital, Paris, F-75020 France; 34 Department of Molecular Medicine, Aarhus University Hospital, Aarhus N, 8200 Denmark; 35 Department of Clinical Medicine, Aarhus University, Aarhus N, 8200 Denmark; 36 Department of Medical Genetics, Oslo University Hospital, Oslo, 0424 Norway; 37 University of Cambridge, Department of Oncology, Addenbrooke’s Hospital, Cambridge, CB2 0QQ UK; 38 Cancer Research UK Cambridge Research Institute, Li Ka Shing Centre, Cambridge, CB2 0RE UK; 39 Nuffield Department of Surgical Sciences, University of Oxford, Oxford, OX1 2JD UK; 40 Faculty of Medical Science, University of Oxford, John Radcliffe Hospital, Oxford, OX1 2JD UK; 41 School of Social and Community Medicine, University of Bristol, Bristol, BS8 2PS UK; 42 Cancer Epidemiology, Nuffield Department of Population Health University of Oxford, Oxford, OX3 7LF UK; 43 Department of Surgical Oncology, Princess Margaret Cancer Centre, Toronto, M5G 2M9 Canada; 44 Department of Radiation Oncology, Icahn School of Medicine at Mount Sinai, New York, 10029 NY USA; 45 Department of Genetics and Genomic Sciences, Icahn School of Medicine at Mount Sinai, New York, 10029-5674 NY USA; 46 Centre for Molecular Oncology, Barts Cancer Institute, Queen Mary University of London, John Vane Science Centre, London, EC1M 6BQ UK; 47 Cancer Epidemiology and Intelligence Division, The Cancer Council Victoria, Melbourne, Victoria, 3004 Australia; 48 Centre for Epidemiology and Biostatistics, Melbourne School of Population and Global Health, The University of Melbourne, Melbourne, VIC 3010 Australia; 49 Division of Urologic Surgery, Brigham and Womens Hospital, Boston, 02115 MA USA; 50 Fundacion Publica Galega de Medicina Xenomica-SERGAS, Grupo de Medicina Xenomica, CIBERER, IDIS, Santiago de Compostela, 15706 Spain; 51 Centre for Research in Environmental Epidemiology (CREAL), Barcelona Institute for Global Health (ISGlobal), Barcelona, 08003 Spain; 52 CIBER Epidemiologia y Salud Publica (CIBERESP), Madrid, 28029 Spain; 53 IMIM (Hospital del Mar Research Institute), Barcelona, 08003 Spain; 54 Universitat Pompeu Fabra (UPF), Barcelona, 08002 Spain; 55 Department of Cancer Epidemiology, Moffitt Cancer Center, Tampa, 33612 USA; 56 Division of Public Health Sciences, Fred Hutchinson Cancer Research Center, Seattle, Washington, 98109-1024 USA; 57 Department of Epidemiology, School of Public Health, University of Washington, Seattle, Washington, 98195 USA; 58 International Hereditary Cancer Center, Department of Genetics and Pathology, Pomeranian Medical University, Szczecin, 70-115 Poland; 59 Faculty of Health and Medical Sciences, University of Copenhagen, Copenhagen, 2200 Denmark; 60 Department of Clinical Biochemistry, Herlev and Gentofte Hospital, Copenhagen University Hospital, Herlev, 2200 Denmark; 61 Division of Clinical Epidemiology and Aging Research, German Cancer Research Center (DKFZ), Heidelberg, D-69120 Germany; 62 German Cancer Consortium (DKTK), German Cancer Research Center (DKFZ), Heidelberg, D-69120 Germany; 63 Division of Preventive Oncology, German Cancer Research Center (DKFZ) and National Center for Tumor Diseases (NCT), Heidelberg, D-69120 Germany; 64 Institute for Human Genetics, University Hospital Ulm, Ulm, 89075 Germany; 65 The University of Texas M. D. Anderson Cancer Center, Department of Genitourinary Medical Oncology, Houston, 77030 TX USA; 66 Cancer Prevention Institute of California, Fremont, 94538 CA USA; 67 Department of Health Research and Policy (Epidemiology) and Stanford Cancer Institute, Stanford University School of Medicine, Stanford, 94305-5101 CA USA; 68 Department of Genetics, Portuguese Oncology Institute of Porto, Porto, 4200-072 Portugal; 69 Biomedical Sciences Institute (ICBAS), University of Porto, Porto, 4050-313 Portugal; 70 Department of Population Sciences, Beckman Research Institute of the City of Hope, Duarte, 91010 CA USA; 71 Ghent University, Faculty of Medicine and Health Sciences, Basic Medical Sciences, Gent, B-9000 Belgium; 72 Department of Surgery, Faculty of Medicine, University of Malaya, Kuala Lumpur, 50603 Malaysia; 73 Department of Urology, University of Washington, Seattle, 98195 WA USA; 74 Institute of Human Genetics, University Medical Center Hamburg-Eppendorf, Hamburg, D-20246 Germany; 75 Molecular Medicine Center, Department of Medical Chemistry and Biochemistry, Medical University, Sofia, 1431 Bulgaria; 76 Department of Oncology, Cross Cancer Institute, University of Alberta, Edmonton, AB T6G 1Z2 Alberta Canada; 77 Division of Radiation Oncology, Cross Cancer Institute, Edmonton, AB T6G 1Z2 Alberta Canada; 78 Molecular Endocrinology Laboratory, Department of Cellular and Molecular Medicine, KU Leuven, BE-3000 Leuven Belgium; 79 Institute of Cancer Sciences, Manchester Cancer Research Centre, University of Manchester, Manchester Academic Health Science Centre, St Mary’s Hospital, Manchester, M13 9WL UK; 80 Genomic Medicine Group, Galician Foundation of Genomic Medicine, Instituto de Investigacion Sanitaria de Santiago de Compostela (IDIS), Complejo Hospitalario Universitario de Santiago, Servicio Galego de Saude, SERGAS, Santiago De Compostela, 15706 Spain; 81 University of California San Diego, Moores Cancer Center, La Jolla, 92037 CA USA; 82 Department of Urology, Erasmus University Medical Center, Rotterdam, 3015 CE The Netherlands; 83 Cancer and Environment Group, Center for Research in Epidemiology and Population Health (CESP), INSERM, University Paris-Sud, University Paris-Saclay, Villejuif, 94807 France; 84 Clinical Gerontology Unit, University of Cambridge, Cambridge, CB2 2QQ UK; 85 Division of Genetic Epidemiology, Department of Medicine, University of Utah School of Medicine, Salt Lake City, 84112 Utah USA; 86 George E. Wahlen Department of Veterans Affairs Medical Center, Salt Lake City, 84148 UT USA; 87 The University of Surrey, Guildford, GU2 7XH Surrey UK; 88 Department of Laboratory Medicine and Pathology, Mayo Clinic, Rochester, 55905 MN USA; 89 Program in Genetic Epidemiology and Statistical Genetics, Department of Epidemiology, Harvard T.H. Chan School of Public Health, Boston, 02115 MA USA

## Abstract

Although genome-wide association studies (GWAS) for prostate cancer (PrCa) have identified more than 100 risk regions, most of the risk genes at these regions remain largely unknown. Here we integrate the largest PrCa GWAS (*N* = 142,392) with gene expression measured in 45 tissues (*N* = 4458), including normal and tumor prostate, to perform a multi-tissue transcriptome-wide association study (TWAS) for PrCa. We identify 217 genes at 84 independent 1 Mb regions associated with PrCa risk, 9 of which are regions with no genome-wide significant SNP within 2 Mb. 23 genes are significant in TWAS only for alternative splicing models in prostate tumor thus supporting the hypothesis of splicing driving risk for continued oncogenesis. Finally, we use a Bayesian probabilistic approach to estimate credible sets of genes containing the causal gene at a pre-defined level; this reduced the list of 217 associations to 109 genes in the 90% credible set. Overall, our findings highlight the power of integrating expression with PrCa GWAS to identify novel risk loci and prioritize putative causal genes at known risk loci.

## Abstract (web summary)

Genome-wide association studies (GWAS) have identified hundreds of genomic risk regions for prostate cancer. Here, the authors perform a transcriptome wide association study (TWAS) by incorporating prostate cancer GWAS with gene expression data to identify potential novel prostate cancer risk loci and possible risk mechanisms.

## Introduction

Prostate cancer (PrCa) affects ~1 in 7 men during their lifetime and is one of the most common cancers worldwide, with up to 58% of risk due to genetic factors^1,2^. Genome-wide association studies (GWAS) have identified over 100 genomic regions harboring risk variants for PrCa which explain roughly one-third of familial risk^3–7^. With few exceptions^8^, the causal variants and target susceptibility genes at most GWAS risk loci have yet to be identified. Multiple studies have shown that PrCa- and other disease-associated variants are enriched near variants that correlate with gene expression levels^9–13^. In fact, recent approaches have integrated expression quantitative trait loci (eQTLs) with GWAS to implicate several plausible genes for PrCa risk (e.g., *IRX4*, *MSMB*, *NCOA4*, *NUDT11*, and *SLC22A3*)^5,14–21^. While overlapping eQTLs and GWAS is powerful, the high prevalence of eQTLs^22^ coupled with linkage disequilibrium (LD) renders it difficult to distinguish the true susceptibility gene from spurious co-localization at the same locus^23^. Therefore, disentangling LD is critical for prioritization and causal gene identification at risk loci.

Gene expression imputation followed by a transcriptome-wide association study^24–26^ (TWAS) has been recently proposed as a powerful approach to prioritize candidate risk genes underlying complex traits. By taking LD into account across SNPs, the resulting association statistics reflect the underlying effect of steady-state gene or alternative splicing expression levels on disease risk^25,27^, which can be used to identify new regions or to rank genes for functional validation at known risk regions^24–28^. Here we perform a multi-tissue transcriptome-wide association study^24–26^ to identify new risk regions and to prioritize genes at known risk regions for PrCa. Specifically, we integrate gene expression data from 48 panels measured in 45 tissues across 4448 individuals with GWAS of prostate cancer from the OncoArray in 142,392 men^29^. Notably, we include alternatively spliced and total gene expression data measured in tumor prostate to identify genes contributing to prostate cancer risk or to continued oncogenesis. We identify 217 gene-trait associations for PrCa with 23 (11) genes identified uniquely using models of alternative spliced (total) expression in tumor. Significant genes were found in 84 independent 1 Mb regions, of which 9 regions are located more than 2 Mb away from any OncoArray GWAS significant variants, thus identifying new candidate risk regions. Second, we use TWAS to investigate genes previously reported as susceptibility genes for prostate cancer identified by eQTL-based analyses. We find a significant overlap with 56 out of 102 previously reported genes assayed in our study also significant in TWAS. Third, we use a novel Bayesian prioritization approach to compute credible sets of genes and prioritize 109 genes that explain at least 90% of the posterior density for association signal at TWAS risk regions. One notable example, *IRX4*, had 97% posterior probability to explain the association signal at its region with the remaining 3% explained by 9 neighboring genes. Overall, our findings highlight the power of integrating gene expression data with GWAS and provide testable hypotheses for future functional validation of prostate cancer risk.

## Results

### Overview of methods

**Fig. 1:**
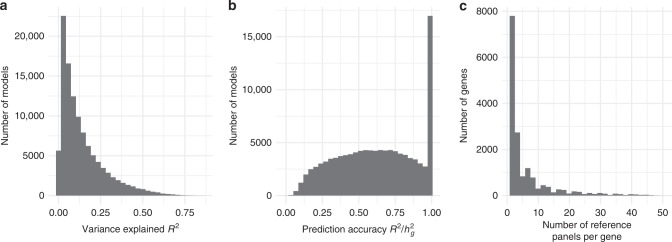
Tissue-specific predictive models for gene expression. **a** Cross-validation prediction accuracy of cis-regulated expression and splicing events (*R*^2^) for all 109,170 tissue-specific models. **b** Normalized prediction accuracy ($R^2/{\rm cis} - h_g^2$) for all 109,170 tissue-specific models. **c** Histogram of the number of reference panels per gene. The majority of genes were heritable in a small number of tissues, but many genes exhibited heritable levels across many tissues

To identify genes associated with PrCa risk, we performed a TWAS using 48 gene expression panels measured in 45 tissues^22,30–36^ integrated with summary data from the OncoArray PrCa GWAS of 142,392 individuals of European ancestry (81,318/61,074 cases/controls; Methods)^29^. We performed the summary-based TWAS approach as described in ref. ^25^ using the FUSION software (Methods). Briefly, this approach uses reference linkage disequilibrium (LD) and reference gene expression panels with GWAS summary statistics to estimate the association between the cis-genetic component of gene expression, or alternative splicing events, and PrCa risk^25^. First, for each panel, FUSION estimated the heritability of steady-state gene and alternative splicing expression levels explained by SNPs local to each gene (i.e., 1 Mb flanking window) using the mixed-linear model (see Methods). Genes with nominally significant (*P* < 0.05) estimates of SNP-heritability (cis-$h_g^2$), are then put forward for training predictive models. Genes with non-significant estimates of heritability are pruned, as they are unlikely to be accurately predicted. Next, FUSION fits predictive linear models (e.g., Elastic Net, LASSO, GBLUP^37^, and BSLMM^38^) for every gene using local SNPs. The model with the best cross-validation prediction accuracy (significant out-of-sample *R*^2^; nominal *P* < 0.05) was used for prediction into the GWAS cohort. This was repeated for all expression datasets, resulting in 109,170 tissue-specific models spanning 15,383 unique genes using total expression and 4990 using alternatively spliced introns for a combined 16,389 unique genes. The average number of models per expression panel was 2228 (Supplementary Data 1). Gene expression measured in normal prostate tissue from GTEx^22^ resulted in only 710 gene models, which can be explained due to smaller sample size (*N* = 87) compared with the average (*N* = 234; Supplementary Data 1). Indeed, the number of gene models per panel was highly correlated with sample size, which implies that statistical power to detect genes with cis-regulatory control is limited by sample size (Supplementary Figure 1). Focusing only on models capturing total gene expression, genes on average had heritable levels of expression in 6.1 different panels (median 3) with 10,628/15,383 genes having heritable expression in at least two panels (Fig. 1). We found *R*^2^ for predictive models was largely consistent across genomic locations, and predominantly affected by the number of non-zero weights used for prediction (Supplementary Figure 2). Predictive power of linear gene expression models is upper-bounded by heritability; thus, we use a normalized *R*^2^ to measure in-sample prediction accuracy (*R*^2^/ cis-$h_g^2$). We found the average *R*^2^/ cis-$h_g^2$ across all tissue-specific models was 65%, which indicates that most of the signal in cis-regulated total expression and alternative splicing levels is captured by the fitted models (Fig. 1). To assess the predictive stability for models of normal prostate gene expression, we compared measured and predicted gene expression for TCGA^36,39^ normal prostate samples using models fitted in GTEx^22^ normal prostate. We found a highly significant replication (mean *R*^2^ = 0.07; *P* = 1.5 × 10^−29^), explaining 41% of in-sample cross-validation *R*^2^ (Supplementary Figure 3), which is consistent with previous out-of-sample estimates^24,25^. We performed a cross-tissue analysis within TCGA and found tumor prostate gene expression models replicated in normal prostate (total expression *R*^2^ = 0.06; splicing *R*^2^ = 0.05; Supplementary Table 1). Given the large number of genes having evidence of genetic control across multiple tissues, we next aimed to measure the similarity of different tissue models (Methods). Across all reference panels for each gene we observed an average *R*^2^ = 0.64 (Supplementary Figure 4). Similarly, when averaging across genes, reference panels displayed an average cross-tissue *R*^2^ = 0.52 (Supplementary Figure 5). Together, these results suggest that trained models predict similar levels of cis-regulated expression on average, despite reference panels measuring expression in different tissues, with varying QC, and differing capture technologies. Next, we performed simulations to measure the statistical power of TWAS under a variety of trait architectures (Supplementary Note 1). Consistent with previous work, we found TWAS to be well-powered at various effect-sizes and heritability levels for gene expression. Importantly, we found no inflation under the null when cis-regulated gene expression has no effect on downstream trait (Supplementary Figure 6).

### TWAS identifies 217 genes associated with PrCa status

**Fig. 2:**
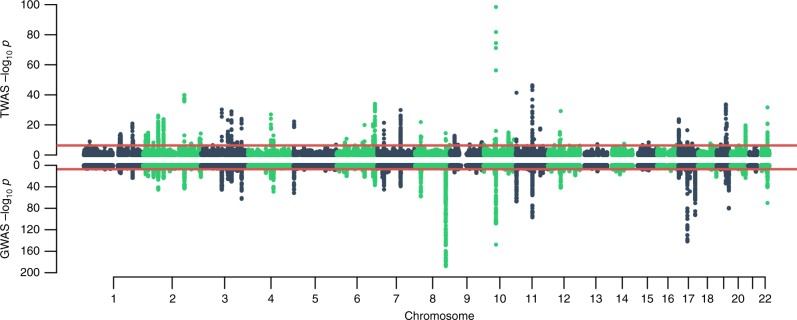
OncoArray PrCa TWAS and GWAS. The top figure is the TWAS Manhattan plot. Each point corresponds to an association test between predicted gene expression with PrCa risk. The red line represents the boundary for transcriptome-wide significance (4.58 × 10^−7^). The bottom figure is the GWAS Manhattan plot where each point is the result of a SNP association test with PrCa risk. The red line corresponds to the traditional genome-wide significant boundary (5 × 10^−8^)

In total, we tested 109,170 tissue-specific gene models of expression for association with PrCa status and observed 892 reaching transcriptome-wide significance (*P*_TWAS_ < 4.58 × 10^−7^; two-tailed *Z*-test), resulting in 217 unique genes, of which 114 were significant in more than one panel (Supplementary Data 2; Fig. 2). On average, we found 18.2 tissue-specific models associated with PrCa per reference expression panel (Supplementary Data 1). In 1 Mb regions with at least 1 transcriptome-wide significant gene, we observed 10.6 tissue-specific associated models on average, and 2.6 associated genes on average, indicating that further refinement of association signal at TWAS risk loci is necessary. To quantify the overlap between non-HLA, autosomal risk loci in the OncoArray PrCa GWAS and our TWAS results, we partitioned GWAS summary data into 1 Mb regions and observed 131 harboring at least one genome-wide significant SNP. Of these, 127/131 overlapped at least one gene model in our data and 68/131 overlapped at least one transcriptome-wide significant gene (Supplementary Figure 7). Associated genes were the closest gene to the top GWAS SNP 20% of the time when using 26,292 RefSeq genes. This result is consistent with previous reports^9,25,26^ and suggests that prioritizing genes based on distance to index SNPs is suboptimal. We found gene model associations were largely consistent, further supporting the predictive stability of models using cis-SNPs (Supplementary Figure 8; Supplementary Note 1). We observed little evidence of prediction accuracy introducing biased results (Supplementary Figure 9; Supplementary Note 1). As a partial control, we compared TWAS results with S-PrediXcan, a related method for predicting gene expression into GWAS summary statistics, using independently trained models and observed a strong correlation (*R* = 0.90; see Supplementary Figure 10; Supplementary Note 1), further supporting the validity of the TWAS approach.

Most of the gene models captured total expression levels in normal tissues, however as a positive control we included models for total expression in tumor prostate tissue (Methods). Predicted expression using tumor prostate models accounted only for 43/217 significant genes compared with 6/217 in normal prostate which is likely due to the large difference in sample size between the original reference panels (Supplementary Data 1). Given this, we found no significant increase in proportion of tumor prostate associated models compared with normal prostate (Fisher’s exact *P* = 0.22). Of the 309 genes with models trained in both reference panels a single shared gene, *MLPH* (OMIM: 606526, a gene whose function is related to melanosome transport^40^), was associated with PrCa risk. In all, 11/43 genes were significant only in tumor prostate models of total expression. We found, 7/11 genes were modeled in other panels but did not reach transcriptome-wide significance while the other 4/11 were not significantly heritable, and thus not testable, in other panels. We also tested models of alternatively spliced introns for association to PrCa risk. We identified predicted expression of alternatively spliced introns in tumor prostate accounted for 68/217 genes, with an average of 2.5 (median 1) alternatively spliced intron associations per significant gene. We next quantified the amount of overlap between results driven from models of alternative splicing events versus models of total gene expression. In all, 23/68 genes were found only in alternatively spliced introns, and 14/23 genes had models of total gene expression but did not reach transcriptome-wide significance. The remaining 9/23 were tested solely in alternatively spliced introns, due to heritability of total gene expression not reaching significance. Together these results emphasize earlier work demonstrating that sQTLs for a gene commonly capture signal independent of eQTLs^41^.

### TWAS analysis increases power to find PrCa associations

Most of the power in the TWAS approach can be attributed to large GWAS sample size. However, two other factors can increase power over GWAS. First, TWAS carries a reduced testing burden compared with that of GWAS, due to TWAS having many fewer genes compared with SNPs. In all, 9/217 genes were located at nine novel independent 1 Mb regions (i.e., no overlapping GWAS SNP), all of which remained significant under a summary-based permutation test (*P* < 0.05/9; Table 1; Supplementary Data 2; Methods). We found this result was stable to increasing region sizes (Supplementary Data 3) and unlikely to be the result of long-range tagging with known GWAS risk (Supplementary Data 4; Supplementary Note 1). We observed increased association signal for SNPs at these regions compared to the genome-wide background after accounting for similar MAF and LD patterns (Supplementary Figure 11), which, together with observed TWAS associations, suggests that GWAS sample size is still a limiting factor in identifying PrCa risk SNPs. As a partially independent check, we performed a multi-tissue TWAS using summary data from an earlier PrCa GWAS (*N* = 49,346)^7^ and found 2 novel regions. We found both regions to overlap a genome-wide significant SNP within 1 Mb in this data further supporting the robustness of TWAS (Supplementary Table 2). Second, we expect to observe increased association signal when expression of a risk gene is regulated by multiple local SNPs^25^. We observed 88/892 instances across 28 genes where TWAS association statistics were stronger than the respective top overlapping GWAS SNP statistics (one-sided Fisher’s exact *P* < 2.2 × 10^−16^; 6.5% higher *χ*^2^ statistics on average). For example, *GRHL3* (OMIM:608317; a gene associated with suppression of squamous cell carcinoma tumors^42^) exhibited stronger signal in TWAS using expression in prostate tumor (*P*_TWAS_ = 9.38 × 10^−10^) compared with the lead SNP signal (*P*_GWAS_ = 1.49 × 10^−5^). Similarly, *POLI* (OMIM:605252, a DNA repair gene associated with mutagenesis of cancer cells^43,44^) resulted in larger TWAS associations (*P*_TWAS_ = 2.29 × 10^−8^) compared with the best proximal SNP (*P*_GWAS_ = 5.44 × 10^−7^).

**Table 1:** Novel risk loci

Gene	Chr	Tx start	Tx end	Exon/exon junction	Expression reference	Best GWAS SNP	Best GWAS P	TWAS P
*GRHL3*	1	24645811	24690970	—	TCGA.PRAD.TUMOR	rs11589294	1.49E−05	9.38E−10*
*GRHL3*				24668763:24669184	TCGA.PRAD_SP.TUMOR			3.08E−07
*FAM83H*	8	49396578	49449526	—	CMC.BRAIN.RNASEQ	rs7831467	3.32E−06	1.66E−07*
*TLE4*	9	82186687	82341796	82189851:82191048	TCGA.PRAD_SP.TUMOR	rs10117770	2.47E−07	2.94E−07*
*TLE4*				—	TCGA.PRAD.TUMOR			1.46E−07*
*TLE4*				82268990:82319698	TCGA.PRAD_SP.TUMOR			1.25E−07*
*TLE4*				82319817:82320804	TCGA.PRAD_SP.TUMOR			2.43E−07*
*TLE4*				82320857:82321662	TCGA.PRAD_SP.TUMOR			2.57E−07*
*TLE4*				82321814:82323033	TCGA.PRAD_SP.TUMOR			2.43E−07*
*TLE4*				82323165:82323508	TCGA.PRAD_SP.TUMOR			2.88E−07*
*TLE4*				82323701:82324538	TCGA.PRAD_SP.TUMOR			2.43E−07*
*TLE4*				82324614:82333637	TCGA.PRAD_SP.TUMOR			2.77E−07*
*TLE4*				82333886:82334961	TCGA.PRAD_SP.TUMOR			8.77E−08*
*TLE4*				82335208:82336656	TCGA.PRAD_SP.TUMOR			3.06E−07*
*TLE4*				82336803:82337366	TCGA.PRAD_SP.TUMOR			1.29E−07*
*TLE4*				82337516:82337874	TCGA.PRAD_SP.TUMOR			2.42E−07*
*TLE4*				82337950:82339952	TCGA.PRAD_SP.TUMOR			2.43E−07*
*STXBP1*	9	130374485	130454995	—	TCGA.PRAD.TUMOR	rs1318074	1.79E−07	2.92E−07*
*STXBP1*				130374719:130413882	TCGA.PRAD_SP.TUMOR			1.88E−07*
*STXBP1*				130413931:130415994	TCGA.PRAD_SP.TUMOR			2.56E−07*
*STXBP1*				130416075:130420654	TCGA.PRAD_SP.TUMOR			2.16E−07*
*STXBP1*				130420730:130422309	TCGA.PRAD_SP.TUMOR			1.39E−07*
*STXBP1*				130422387:130423381	TCGA.PRAD_SP.TUMOR			4.10E−07*
*STXBP1*				130423484:130425484	TCGA.PRAD_SP.TUMOR			2.22E−07*
*STXBP1*				130425632:130427526	TCGA.PRAD_SP.TUMOR			2.75E−07*
*STXBP1*				130428575:130430359	TCGA.PRAD_SP.TUMOR			2.51E−07*
*STXBP1*				130430466:130432177	TCGA.PRAD_SP.TUMOR			2.51E−07*
*STXBP1*				130432237:130434330	TCGA.PRAD_SP.TUMOR			2.06E−07*
*STXBP1*				130434395:130435460	TCGA.PRAD_SP.TUMOR			1.47E−07*
*STXBP1*				130435540:130438083	TCGA.PRAD_SP.TUMOR			2.60E−07*
*STXBP1*				130438221:130438923	TCGA.PRAD_SP.TUMOR			3.15E−07*
*STXBP1*				130439032:130440710	TCGA.PRAD_SP.TUMOR			1.98E−07*
*RP11-57H14.2*	10	114710405	114711634	—	GTEx.Esophagus_Muscularis	rs11196152	1.61E−07	1.40E−07*
*RP11-57H14.2*				—	GTEx.Lung			9.81E−08*
*RP11-57H14.2*				—	GTEx.Nerve_Tibial			3.29E−08*
*RP11-57H14.2*				—	GTEx.Pituitary			1.11E−07*
*RP11-57H14.2*				—	GTEx.Thyroid			3.40E−07*
*RP11-57H14.2*				—	GTEx.Whole_Blood			1.97E−08*
*TM7SF3*	12	27124505	27167339	27129290:27132717	TCGA.PRAD_SP.TUMOR	rs16931510	3.06E−07	2.27E−07*
*POLI*	18	51795773	51824604	—	NTR.BLOOD.RNAARR	rs11083046	5.44E−07	2.29E−08*
*POLI*				—	GTEx.Adipose_Subcutaneous			1.92E−07*
*POLI*				—	GTEx.Artery_Aorta			2.25E−07*
*POLI*				—	GTEx.Artery_Tibial			1.54E−07*
*POLI*				—	GTEx.Brain_Cerebellar_Hemisphere			1.63E−07*
*POLI*				—	GTEx.Brain_Cerebellum			1.56E−07*
*POLI*				—	GTEx.Brain_Putamen_basal_ganglia			3.54E−07*
*POLI*				—	GTEx.Breast_Mammary_Tissue			3.20E−07*
*POLI*				—	GTEx.Cells_EBV-transformed_lymphocytes			1.63E−07*
*POLI*				—	GTEx.Colon_Sigmoid			4.86E−08*
*POLI*				—	GTEx.Esophagus_Gastroesophageal_Junction			1.99E−07*
*POLI*				—	GTEx.Esophagus_Mucosa			2.15E−07*
*POLI*				—	GTEx.Esophagus_Muscularis			2.08E−07*
*POLI*				—	GTEx.Heart_Atrial_Appendage			1.36E−07*
*POLI*				—	GTEx.Lung			4.39E−07*
*POLI*				—	GTEx.Nerve_Tibial			9.62E−08*
*POLI*				—	GTEx.Spleen			2.74E−07*
*POLI*				—	GTEx.Testis			1.43E−07*
*POLI*				—	GTEx.Thyroid			2.34E−08*
*POLI*				—	GTEx.Whole_Blood			4.11E−07*
*POLI*				—	METSIM.ADIPOSE.RNASEQ			3.89E−07*
*POLI*				—	YFS.BLOOD.RNAARR			2.94E−07*
*POLI*				51807273:51809207	TCGA.PRAD_SP.TUMOR			4.47E−07*
*KDSR*	18	60994959	61034743	—	GTEx.Adipose_Subcutaneous	rs1541296	3.98E−07	4.20E−07*
*UQCC1*	20	33935075	33954360	33935075:33954360	TCGA.PRAD_SP.TUMOR	rs7280	3.98E−07	4.40E−07*

TWAS associations that did not overlap a genome-wide significant SNP (i.e., ±1 Mb transcription start site). Study denotes the original expression panel used to fit weights. *P*-value for TWAS computed under the null of no association between gene expression levels and PrCa risk under a Normal (0, 1) distribution. An asterisk (*) indicates associations that are significant (*P* < 0.05/9) under a permutation test

### TWAS replicates previously reported genes

**Fig. 3:**
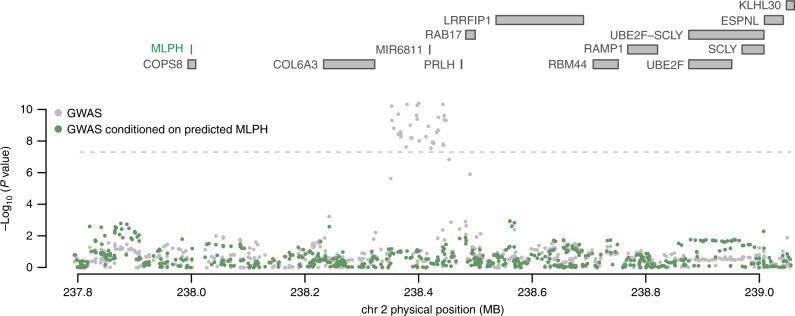
Predicted expression of *MLPH* explains majority of GWAS signal at its genomic region. Each point corresponds to the association between SNP and PrCa status. Gray points indicate the marginal association of a SNP with PrCa status (i.e., GWAS association). Green points indicate the association of the same SNPs with PrCa after conditioning on predicted expression of *MLPH* using models trained from normal prostate (GTEx) and tumor prostate (TCGA). The dashed gray line corresponds to the genome-wide significant threshold (i.e., *P* = 5 × 10^−8^). *MLPH* was discussed in previous works as a possible susceptibility gene for PrCa. Association between total expression of *MLPH* and PrCa risk was transcriptome-wide significant in normal and tumor prostate tissue

We next sought to quantify the extent of overlapping results between TWAS and previous studies that integrated eQTL data measured in normal and tumor prostate tissues at PrCa risk regions (Methods; Supplementary Table 3)^5,14–20^. We considered only autosomal, non-HLA genes which resulted in 130 previously reported genes. We found a significant overlap between reported genes, with 102/130 assayed in our study and 56/102 reaching transcriptome-wide significance in at least one of our panels (Fisher’s exact *P* < 2.2 × 10^−16^; Supplementary Table 3, Supplementary Data 5). For example, *MLPH* was reported in 4/8 studies. We found significant associations suggesting that decreased expression of *MLPH* in normal and tumor prostate tissue increases risk for PrCa (e.g., GTEx prostate *MLPH Z*_TWAS_ = −5.80; *P*_TWAS_ = 6.69 × 10^−9^; TCGA prostate *Z*_TWAS_ = −6.77; *P*_TWAS_ = 1.25 × 10^−11^). Predicted *MLPH* in tumor prostate remained significant under permutation, which suggests that chance co-localization with GWAS risk is unlikely (Supplementary Data 2). To assess the amount of residual association signal due to genetic variation in the GWAS risk region after accounting for predicted expression of *MLPH*, we performed a summary-based conditional analysis (Methods). We found *MLPH* to explain most of the signal at its region (lead SNP *P*_GWAS_ = 4.03 × 10^−11^; conditioned on *MLPH* lead SNP *P*_GWAS_ = 1.13 × 10^−3^; Fig. 3). Our findings are consistent with recent work that found decreased expression levels of *MLPH* to be associated with increased PrCa risk^45^. Despite previous eQTL data focusing on normal and tumor prostate tissue, we observed associations in 45 expression panels overlapping the 56 observed genes in total, underscoring earlier works demonstrating the consistency of cross-tissue cis-regulatory effects^46^.

### Prioritization pinpoints a single gene for most risk regions

TWAS genes are indicative of association and do not necessarily reflect causality (e.g., due to co-regulation at the same region). To prioritize genes at regions with multiple TWAS signals (Fig. 2), we used a Bayesian formulation to estimate 90%-credible gene sets (Methods). We found 109 unique genes across 84 non-overlapping 1 Mb regions comprising our 90% credible sets (Supplementary Data 6, 7). In all, 68/84 credible sets contained either a single gene or the same gene in multiple tissues. The average number of unique genes per credible set was 1.29 (median 1). We observed that 28/109 prioritized genes were previously reported in eQTL analyses^5,14–20^, which supports the hypothesis that TWAS followed by Bayesian prioritization refines associations to relevant disease genes. For example, *MLPH* was the sole gene defining its region’s 90% credible set with a posterior probability of 94%. Similarly, *SLC22A3* (OMIM: 604842; a gene involved in poly-specific organic cation transporters^47^ and previously implicated in PrCa risk^18^) exhibited >94% posterior probability to be causal.

### Prostate tissue genes have largest average effect

**Fig. 4:**
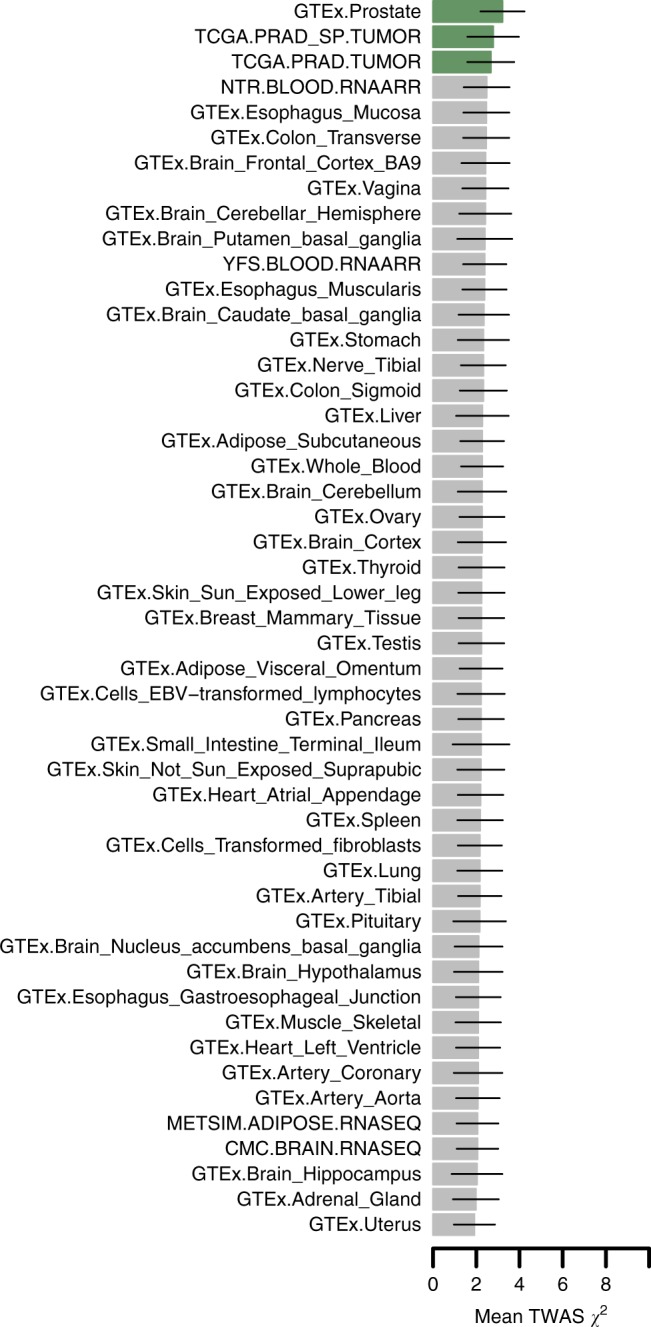
Average TWAS association statistics for genes predicted in each expression panel. Each bar plot corresponds to the average TWAS association statistic using all gene models from a given expression reference panel. Lines represent 1 standard-deviation estimated using the median absolute deviation under normality assumptions. Normal and tumor prostate tissues are marked in green. All other tissues are marked in gray

Given the large number of significant associations observed for non-prostate tissues in our data, we wanted to quantify which tissue is most relevant for PrCa risk. We first grouped TWAS PrCa associations into prostate/non-prostate and tested for enrichment in normal and tumor prostate expression models. Predicted expression and splicing events in normal and tumor prostate made up 221/892 associations with PrCa (Supplementary Data 2) which was highly significant compared to the grouping of all other tissues (Fisher’s exact *P* = 7.3 × 10^−9^). This measure only quantifies the total amount of observed associations and neglects average association strength. Next, we computed the mean TWAS association statistic using all genes predicted from each expression reference panel (Fig. 4). We observed the largest average TWAS associations in genes predicted from normal and tumor prostate tissue, which reaffirms our intuition of expression and splice events in prostate being the most relevant for PrCa risk. We re-ranked mean associations using only genes found to be transcriptome-wide significant and observed a similar ordering with total expression in normal prostate ranked highest (average *χ*^2^ = 176.2; Supplementary Figure 12).

## Discussion

Prostate cancer is a common male cancer that is expected to affect more than 180,000 men in the United States in 2017 alone^48^. While GWAS has been successful in localizing risk for PrCa due to genetic variation, the underlying susceptibility genes remain elusive. Here we have presented results of a transcriptome-wide association study using the OncoArray PrCa GWAS summary statistics for over 142,000 case/control samples. This approach utilizes imputed expression levels and splicing events in the GWAS samples to identify and prioritize putative susceptibility genes. We identified 217 genes whose expression is associated with PrCa risk. These genes localized at 84 genomic regions, of which nine regions do not overlap with a genome-wide significant SNP in the OncoArray GWAS. We found 23 genes using predictive models for alternatively spliced introns in tumor prostate, which supports the its role in continued risk for tumor oncogenesis. A large fraction of identified genes was confirmed in earlier work, with 56 genes previously reported in eQTL/PrCa GWAS overlap studies. We used a novel Bayesian prioritization approach to refine our associations to credible sets of 109 genes with statistical evidence of causality under standard assumptions. Our results provide a functional map for PrCa risk which can be explored for follow-up and validation.

In this study, we compared our reported TWAS results with genes identified in previous works focusing on expression measured in normal and tumor prostate tissue. Several of these studies considered an eQTL and GWAS risk SNP to overlap if they are in linkage at a specified threshold. While these approaches are sound, they may be limited in statistical power for several reasons. First, if multiple local SNPs independently contribute to risk, overlap studies relying only on the top risk SNP will lose power. Second, earlier overlap studies used thresholds for association signal (i.e., GWAS *P* < 5 × 10^−8^) and linkage strength (i.e., LD > 0.5) to consider pairs of SNPs for evidence of expression influencing risk of PrCa. TWAS is largely agnostic to both issues as it jointly considers all SNPs in the region, regardless of reported GWAS association strength. However, when expression of a risk gene is regulated by a single causal SNP, we expect TWAS and earlier overlap approaches to have similar levels in power^25^.

Previous works have strongly implicated expression of certain genes in PrCa risk that were not assayed in our study (e.g., MSMB^18,49^) due to non-significant heritability estimates. TWAS operates by fitting predictive linear models of gene expression based on local genotype data, followed by prediction into large cohorts and subsequent association testing. Expression of genes that are not significantly heritable at current sample sizes are not included in the pipeline. This is the consequence of heritability providing an upper bound on the predictive accuracy under a linear model for genotype; therefore, if a gene has undetectable heritability at a given sample size, it will be difficult to predict using linear combinations of SNPs. To compute TWAS weights for normal prostate tissue, we used samples collected in the GTEx v6 panel (*n* = 87). Thus, our inability to detect heritable levels of gene expression can be explained due to the relatively small number of samples compared with other tissues. Indeed, previous work has shown a strong correlation between sample size in expression panels and the number of identified eGenes^27^; therefore, as sample size increases for relevant tissues, we expect the number of genes included in the TWAS framework to increase. TWAS will lose power in situations where gene expression is a nonlinear function of local SNPs, or when trans (or distal) regulation is a major component in modulating expression levels.

We conclude with several caveats and possible future directions. First, while TWAS associations are consistent with models of steady-state gene expression levels altering risk for PrCa, they may be the result of confounding^25,26^. Imputed gene expression levels are the result of weighted linear combinations of SNPs, many of which may tag non-regulatory mechanisms driving risk and result in inflated association statistics. Second, our results relied on validating prediction models using multiple approaches: within-reference methods (i.e., cross-validation), cross-reference methods (e.g., GTEx into TCGA), and external-reference methods (i.e., 1000 Genomes predictive stability). While results from these approaches support our models generalizing out-of-sample, we still lack within-GWAS replication of predictive models. Third, since genes with eQTLs are common, associations may be the result of chance co-localization between eQTLs and PrCa risk. Finally, we note recent work has extended TWAS-like methods to expose regulatory mechanisms for susceptibility genes by incorporating chromatin information^50^. An extension to our work would be to pinpoint chromatin variation regulating expression levels at identified risk genes, thus describing a richer landscape of the molecular cascade where SNP → chromatin → expression → PrCa risk.

## Methods

### OncoArray GWAS summary statistics

Genome-wide association summary statistics for the OncoArray PrCa study were obtained from ref. ^29^. Summary statistics were computed using a fixed-effect meta-analysis for 142,392 total samples of European ancestry from the OncoArray (81,318/61,074 cases/controls), UK stage 1 (1854/1894) and UK stage 2 (3706/3884), CaPS 1 (474/482) and CaPS 2 (1458/512), BPC3 (2068/3011), NCI PEGASUS (4600/2941) and iCOGS (20,219/20,440). The initial summary data contained association statistics for 19,726,430 variants. We filtered out summary statistics for SNPs with MAF <0.01 and any SNPs with ambiguous alternative alleles (e.g., A → T; C → G; or vice-versa). Finally, we kept only SNPs with rsIDs defined by dbSNP144. Our QC pipeline resulted in association statistics at 10,516,237 SNPs for downstream TWAS analyses.

### Previous prostate-tissue eQTL studies

We collected previous studies that investigated the overlap of eQTLs in normal and tumor prostate tissue at known PrCa risk loci^5,14–20^. We compared TWAS statistics versus reported eQTL overlap results as aggregated in refs. ^14,15^. Across these studies, overlap of eQTLs and PrCa risk loci are computed by one of two possible methods. The first method tests known PrCa risk SNPs for association with expression levels of nearby genes/transcripts. The second method takes a two-step approach. First, genes nearby PrCa risk loci are tested for harboring eQTLs at some significance level. Next, genes with identified eQTL SNPs are tested to be in LD with known PrCa risk variants at some level (e.g., *r*^2^ > 0.5).

### Reference gene expression data and predictive models of expression

We downloaded the FUSION software (see URLs) along with its prepackaged weights for gene expression data. FUSION is an R package that implements the TWAS scheme described in ref. ^25^. Weights for gene expression measured using RNA sequencing data were obtained from the CommonMind Consortium^30^ (dorsolateral prefrontal cortex, *n* = 452), the Genotype-Tissue Expression Project^22^ (GTEx; 44 tissues; *n* = 449), the Metabolic Syndrome in Men study^32,33^ (adipose, *n* = 563), and The Cancer Genome Atlas (TCGA; prostate adenocarcinoma, *n* = 483)^39^. Expression microarray data were obtained from the Netherlands Twins Registry^35^ (NTR; blood, *n* = 1247), and the Young Finns Study^31,34^ (YFS; blood, *n* = 1264). All non-TCGA expression panel individuals were PrCa controls. Detailed description of quality control procedures on measured gene expression and genotype information for all non-TCGA reference panels are described in refs. ^25,27^. TCGA genotype, gene expression, and exon-junction data for 525 samples were downloaded using the Broad GDAC FireHose version 2016_1_28 (see URLs). Genotypes were imputed to the Haplotype Reference Consortium^51^ and restricted to well-imputed (INFO > 0.9) HapMap3^52^ sites. Genes (exon junctions) missing in more than half of samples were removed. RPKM and log-adjusted gene expression levels were estimated in a generalized linear model controlling for three gene expression PCs. The estimated log-abundances were quantile-normalized and inverse-normal rank-normalized. We estimated alternatively spliced introns using the software MapSplice version 2 (see URLs). A total of 482 samples passed quality control procedures in both genotype and gene expression data. We note that batch effects from measurement biases (e.g., RNA-degradation) should be uncorrelated with SNPs local to a gene body definition, and therefore not impact prediction accuracy. By maximizing the sample size, predictive power when using cis-SNPs should increase and be largely unbiased. This is evidenced by the fact that models are largely stable across and within TCGA PRAD datasets (Supplementary Table 1).

We filtered genes that did not exhibit cis-genetic regulation at current samples sizes by keeping only genes with nominally significant (*P* < 0.05) estimates of cis-SNP heritability (cis-$h_g^2$), which resulted in 117,459 total tissue-gene pairs from 17,023 unique genes. We refrain from reporting genes from the HLA region due to complicated LD patterns.

To train predictive models, FUSION defines gene expression for *n* samples (**y**_GE_) as a linear function of *p* SNPs (**X**) in a 1 Mb region flaking the gene as

$${\mathbf{y}}_{{\mathrm{GE}}} = {\mathbf{C\beta}} + {\mathbf{Xw}}_{{\mathrm{GE}}} + {\bf{\epsilon }},$$

where **w**_GE_ are the *p* SNP weights, **Cβ** are covariates (e.g., sex, age, genotype principal components, genotyping platform, and PEER factors) and their effects, and $\epsilon$ is random environmental noise. FUSION estimated weights for expression of a gene in a tissue using multiple penalized linear models. Generally, FUSION optimizes for

$$\left[ {\begin{array}{*{20}{c}} {{\hat{\mathbf w}}_{{\mathrm{GE}}}} \\ {{\hat{\mathbf \beta }}} \end{array}} \right] = \arg \mathop {{\min }}\limits_{{\mathbf{w}}_{{\mathrm{GE}}},{\mathbf{\eta }}} \|{\mathbf{y}}_{{\mathrm{GE}}} - {\mathbf{Xw}}_{{\mathrm{GE}}} - {\mathbf{C\beta }}\|_2^2 + f\left( {{\mathbf{w}}_{{\mathrm{GE}}}} \right),$$

where *f*(**w**_GE_) is a parameterized penalty function specific to each model (e.g., GBLUP^37^, LASSO, the Elastic Net). The exception to this optimization criterion is the Bayesian sparse linear mixed model (i.e., BSLMM)^38^ which fits the posterior mean for **w**_GE_ using MCMC in the GEMMA v 0.94 software (see URLs) to obtain weights. To determine which model has the best prediction accuracy for a given gene-tissue pair, FUSION computes out-of-sample *R*^2^ by performing fivefold cross-validation for each model. We compute the normalized prediction accuracy for a gene as ${\mathrm{min}}\left( {\frac{{R^2}}{{h_g^2}},1} \right)$. Weights from the model with the largest *R*^2^ that was also nominally non-zero (*P* < 0.05) were used to compute TWAS association statistics. This resulted in a final tally of 109,170 tissue-specific models at 16,389 unique genes.

### Cis-heritability of gene expression

FUSION reports the estimated SNP-heritability (i.e., $h_g^2$) for measured gene expression levels explained by SNPs in the cis-region (1 Mb region surrounding the TSS). This is modeled under a mixed-linear model as

$${\mathrm{var}}\left( {{\mathbf{y}}\prime _{{\mathrm{GE}}}} \right) = {\mathbf{A}}{{\sigma }}_{{g}}^2 + {\mathbf{I}}{{\sigma }}_{{e}}^2,$$

where ${\mathbf{y}}\prime _{{\rm GE}}$ is the residual gene expression after regressing out fixed-effect covariates **C**, **A** is the estimated kinship matrix from SNPs in the cis-region and ${\mathrm{\sigma }}_g^2$ (${\mathrm{\sigma }}_e^2$) is the variance explained by the cis-SNPs (environment). SNP-heritability is then defined to be ratio of genotypic variance and total trait variance as, $h_g^2 = \frac{{{\mathrm{\sigma }}_g^2}}{{{\mathrm{\sigma }}_g^2 + {\mathrm{\sigma }}_e^2}}$. Variance parameters are estimated using the AI-REML algorithm implemented in GCTA v1.26 (see URLs) with the top 3 genotypic principal components, sex, age, genotyping platform, and PEER factors as covariates.

### Measuring cross-tissue similarity in predicted expression

We took an unbiased approach to identify susceptibility genes for PrCa by using gene expression panels measured in various tissues. To quantify how similar predicted expression levels are for the same gene across different tissues, we measured the squared Pearson correlation (*R*^2^). This value represents how well predicted expression from one tissue predicts expression in another tissue. To dissect similarities and differences of tissue-specific models, the ideal scenario would be to inspect effects at individual SNPs defining the models. In practice this is not possible due to predictive models not including the same set of SNPs due to QC and technological differences in the original studies. Therefore, as a proxy we predict gene expression into the 489 samples of European ancestry from 1000 Genomes^53^ and compute *R*^2^ across shared genes for pairs of tissues (Supplementary Note 1).

### Transcriptome-wide association study using GWAS summary statistics

FUSION estimates the strength of association between predicted expression of a gene and PrCa (z_TWAS_) as function of the vector of GWAS summary *Z*-scores at a given cis locus z_GWAS_ (i.e., vector of SNP association Wald statistics) and the LD-adjusted weights vector learned from the gene expression data **w**_GE_ as

$${\rm z}_{{\rm TWAS}} = \frac{{{\mathbf{{w}}}_{{\rm GE}}^\prime {\mathbf{{z}}}_{{\rm GWAS}}}}{{\sqrt {{\mathop{\rm{var}}} ({\mathbf{{w}}}_{{\rm GE}}^\prime {\mathbf{{z}}}_{{\rm GWAS}})} }} = \frac{{{\mathbf{{w}}}_{{\rm GE}}^\prime {\mathbf{{z}}}_{{\rm GWAS}}}}{{\sqrt {{\mathbf{{w}}}_{{\rm GE}}^\prime {\mathbf{{Vw}}}_{{\rm GE}}} }},$$

where **V** is a correlation matrix across SNPs at the locus (i.e., LD) and “‘” indicates transpose. A *P*-value for z_TWAS_ is obtained using a two-tailed test under *N*(0,1). In this work, we estimated **V** using 489 samples of European ancestry in 1000 Genomes^53^. To account for the large number of hypotheses tested, we perform Bonferroni correction at *α* = 0.05/*M*, where *M* = 109,170 is the number of predictive models, which is conservative as many gene models are correlated. As reported by ref. ^25^, there may be inflation at GWAS risk loci, due to chance co-varying of SNP effects between expression and PrCa. The same work described a permutation procedure that assesses likelihood of observing association by chance conditioned on GWAS signal. The algorithm works by permuting the eQTL weights **w**_GE_ while keeping **z**_GWAS_ fixed and computing **z**_TWAS_,_perm_. FUSION implements an adaptive procedure that stops once enough scores (i.e. |**z**_TWAS_,_perm_|≥|**z**_TWAS_|) have been observed such that the empirical null cannot be rejected at a specified level. We define novel risk regions as a flanking region around a transcriptome-wide significant gene (splicing event; *P*_TWAS_ < 4.58 × 10^−7^; two-tailed *Z*-test) that does not harbor a genome-wide significant SNP (*P*_GWAS_ < 5 × 10^−8^; two-tailed *Z*-test). We consider 2 Mb windows by default (i.e. TSS ± 1 Mb) and show that the results are robust to the choice of window size (Supplementary Data 3).

### GWAS analyses conditional on predicted expression

To assess the extent of residual association of SNP with PrCa risk after accounting for predicted gene expression levels, FUSION estimates conditional SNP association scores using GWAS summary statistics. Namely, define **V** as LD for SNPs in the region, **V**_**GE**_ as the correlation between predicted expression levels, and **C** as the correlation between SNPs and predicted expression. The least-squares estimates of **z**_GWAS_|**z**_TWAS_ are determined by,

$${\mathbf{z}}_{{\mathrm{GWAS}}}|{\mathbf{z}}_{{\mathrm{TWAS}}} = {\mathbf{z}}_{{\mathrm{GWAS}}} - {\mathbf{CV}}_{{\mathbf{GE}}}^{ - 1}{\mathbf{z}}_{{\mathrm{TWAS}}}.$$

The variance of the residual association strength is given by,

$${\mathrm{var}}\left[ {{\mathbf{z}}_{{\mathrm{GWAS}}}|{\mathbf{z}}_{{\mathrm{TWAS}}}} \right] = {\mathrm{var}}\left[ {{\mathbf{z}}_{{\mathrm{GWAS}}}} \right] - {\mathrm{var}}\left[ {{\mathbf{CV}}_{{\mathbf{GE}}}^{ - 1}{\mathbf{z}}_{{\mathrm{TWAS}}}} \right] = {\mathbf{V}} - {\mathbf{CV}}_{{\mathbf{GE}}}^{ - 1}{\mathbf{C}}^{\prime}.$$

This results in the final conditional association score for the *i*th SNP as,

$${{z}}_{{i}} = \left[ {{\mathbf{z}}_{{\mathrm{GWAS}}} - {\mathbf{CV}}_{{\mathbf{GE}}}^{ - 1}{\mathbf{z}}_{{\mathrm{TWAS}}}} \right]_{{i}}/\surd {\mathrm{diag}}\left[ {{\mathbf{V}} - {\mathbf{CV}}_{{\mathbf{GE}}}^{ - 1}{\mathbf{C}}^\prime } \right]_{{{ii}}}.$$

### Bayes factors and posterior inference of causal genes

Complex correlations between predicted expression levels at a given region can yield multiple associated genes in TWAS (Fig. 2). Thus, for the vast majority of risk regions it remains unclear which gene is causally influencing PrCa risk. Here, modeling under the assumption of a single causal gene per risk region and relying on the central limit theorem for normality, we can compute the Bayes Factor that the *i*th gene in a region is causal as,

$${\rm BF}_i = \frac{{N\left( {{\rm {z}}_{{\mathrm{TWAS}},{{i}}}|0,1 + n\sigma _\alpha ^2} \right)}}{{N\left( {{\rm {z}}_{{\mathrm{TWAS}},{{i}}}|0,1} \right)}} = \left( {1 + n\sigma _\alpha ^2} \right)^{ - 1/2}\exp \left( {\frac{{{\rm {z}}_{{\mathrm{TWAS}},{{i}}}^2}}{2}\frac{{n\sigma _\alpha ^2}}{{1 + n\sigma _\alpha ^2}}} \right),$$

where ${\rm z}_{{\mathrm{TWAS}},{{i}}}^2$ is the squared TWAS association statistic for the *i*th gene, *n* is the GWAS sample size, and $\sigma _\alpha ^2$ is prior effect-size variance for gene expression on PrCa risk (Supplementary Note 1). This model is structurally similar in form to earlier works^54–56^ describing Bayes Factors for fine mapping SNPs at GWAS risk regions. The important distinction is that here, we formulate a Bayes Factor for genes at TWAS risk regions. The Bayes Factor for each gene quantifies the amount of evidence in favor of the causal model (*i*th gene drives risk) versus the null (*i*th gene has no causal effect). We extend individual Bayes Factors for *k* genes at a PrCa risk region to compute the posterior probability that a gene is causal as,

$$\Pr \left( {{\mathrm{gene}}\,i\,{\mathrm{is}}\,{\mathrm{causal}}|{\mathbf{{z}}}_{{\mathrm{TWAS}}},n\sigma _\alpha ^2} \right) = \frac{{{\rm BF}_{{i}}}}{{\mathop {\sum }\nolimits_{\mathrm{k}} {\rm {BF}}_{\mathrm{k}}}}.$$

Equipped with our definition of posterior probability for each gene being causal, we define *ρ*-credible gene sets for a PrCa risk region. Formally, a set of indices $i \in I$ defines a *ρ*-credible gene set if

$$\rho = \mathop {\sum}\limits_{i \in I} {\Pr \left( {{\mathrm{gene}}\,i\,{\mathrm{is}}\,{\mathrm{causal|}}{\mathbf{{z}}}_{{\mathrm{TWAS}}},\,n\sigma _\alpha ^2} \right).}$$

For a fixed *ρ* we optimize over k genes at a region by greedily adding genes until the total density is at least *ρ*.

To ensure that our *ρ*-credible sets are well-calibrated we performed simulations by predicting expression levels into 489 samples of European ancestry from 1000 Genomes^53^ and estimating the local correlation structure to sample TWAS *Z*-scores directly (Supplementary Note 1). Under the assumption of a single causal gene at a risk region, we sampled TWAS *Z*-scores for 1000 independent regions. We then performed Bayesian prioritization at each region and computed *ρ*-credible sets for various levels of *ρ* while counting the proportion of causal genes identified across all simulations.

### Pathway analyses

To determine which pathways may be enriched with genes identified from our Bayesian prioritization approach, we used the R package GOseq^57^ which internally links gene identifiers to GO terms (GO db: 2017-09-02). We categorized all 16,389 genes into prioritized/not-prioritized and ran the analysis using custom R scripts linking GOseq. GOseq obtains *P*-values for overrepresented genes using the Wallenius approximation to the non-central hypergeometric distribution. We limited analysis to Gene Ontology Biological Pathways (GO:BP). GOSeq drops genes without GO annotations from analysis. We observed 4711 genes dropped from analyses resulting in 11,678 genes put forward for enrichment tests (Supplementary Data 8; Supplementary Note 1).

### URLs

1000 Genomes Phase3: http://www.internationalgenome.org/

Fire Hose v2016_1_28: http://gdac.broadinstitute.org/

FUSION: http://gusevlab.org/projects/fusion/

GCTA v1.26: http://cnsgenomics.com/software/gcta/

GEMMA v0.94: http://www.xzlab.org/software.html

GOseq v1.26: http://bioinf.wehi.edu.au/software/goseq/

MapSplice v2: http://www.netlab.uky.edu/p/bioinfo/MapSplice2

PLINK v1.9: https://www.cog-genomics.org/plink2/

OncoArray: https://epi.grants.cancer.gov/oncoarray/

## Electronic supplementary material

Supplementary Information

Description of Additional Supplementary Files

Supplementary Data 1

Supplementary Data 2

Supplementary Data 3

Supplementary Data 4

Supplementary Data 5

Supplementary Data 6

Supplementary Data 7

Supplementary Data 8

## Data Availability

Complete TWAS and fine-mapping results are available at http://github.com/bogdanlab/prca_twas/. OncoArray PrCa GWAS summary data used in this study are available at http://practical.icr.ac.uk/blog/. Relevant TCGA data are available from Broad Firehouse at http://gdac.broadinstitute.org. FUSION software, weights/models, and reference LD are available at http://gusevlab.org/projects/fusion/.
