# Author Correction: Large-scale transcriptome-wide association study identifies new prostate cancer risk regions

**DOI:** 10.1038/s41467-018-08108-7

**Published:** 2019-01-08

**Authors:** Nicholas Mancuso, Simon Gayther, Alexander Gusev, Wei Zheng, Kathryn L. Penney, The PRACTICAL consortium, Brian E. Henderson, Sara Benlloch, Fredrick R. Schumacher, Ali Amin Al Olama, Kenneth Muir, Sonja I. Berndt, David V. Conti, Fredrik Wiklund, Stephen Chanock, Victoria L. Stevens, Catherine M. Tangen, Jyotsna Batra, Judith Clements, Henrik Gronberg, Nora Pashayan, Johanna Schleutker, Demetrius Albanes, Stephanie Weinstein, Alicja Wolk, Catharine West, Lorelei Mucci, Géraldine Cancel-Tassin, Stella Koutros, Karina Dalsgaard Sorensen, Lovise Maehle, David E. Neal, Freddie C. Hamdy, Jenny L. Donovan, Ruth C. Travis, Robert J. Hamilton, Sue Ann Ingles, Barry Rosenstein, Yong-Jie Lu, Graham G. Giles, Adam S. Kibel, Ana Vega, Manolis Kogevinas, Jong Y. Park, Janet L. Stanford, Cezary Cybulski, Børge G. Nordestgaard, Hermann Brenner, Christiane Maier, Jeri Kim, Esther M. John, Manuel R. Teixeira, Susan L. Neuhausen, Kim De Ruyck, Azad Razack, Lisa F. Newcomb, Davor Lessel, Radka Kaneva, Nawaid Usmani, Frank Claessens, Paul A. Townsend, Manuela Gago-Dominguez, Monique J. Roobol, Florence Menegaux, Kay-Tee Khaw, Lisa Cannon-Albright, Hardev Pandha, Stephen N. Thibodeau, David J. Hunter, Peter Kraft, Zsofia Kote-Jarai, Rosalind Eeles, Matthew Freedman, Christopher Haiman, Bogdan Pasaniuc

**Affiliations:** 1 Department of Pathology and Laboratory Medicine, David Geffen School of Medicine, University of California, Los Angeles, Los Angeles, 90095 CA USA; 2 The Center for Bioinformatics and Functional Genomics, Cedars-Sinai Medical Center, Los Angeles, 90048 CA USA; 3 Dana Farber Cancer Institute, Boston, 02215 MA USA; 4 Division of Epidemiology, Department of Medicine, Vanderbilt Epidemiology Center, Vanderbilt-Ingram Cancer Center, Vanderbilt University School of Medicine, Nashville, 37232 TN USA; 5 Department of Epidemiology, Harvard T.H. Chan School of Public Health, Boston, 02115 MA USA; 6 Channing Division of Network Medicine, Department of Medicine, Brigham and Women’s Hospital/Harvard Medical School, Boston, 02115 MA USA; 7 Division of Genetics and Epidemiology, The Institute of Cancer Research, London, SW7 3RP UK; 8 Royal Marsden NHS Foundation Trust, London, SW3 6JJ UK; 9 Department of Medical Oncology, Dana-Farber Cancer Institute and Harvard Medical School, Boston, 02215 MA USA; 10 Department of Preventive Medicine, Norris Comprehensive Cancer Center, Keck School of Medicine, University of Southern California, Los Angeles, 90015 CA USA; 11 Department of Human Genetics, David Geffen School of Medicine, University of California, Los Angeles, Los Angeles, 90095 CA USA; 12 Bioinformatics Interdepartmental Program, University of California, Los Angeles, Los Angeles, 90095 CA USA; 13 Centre for Cancer Genetic Epidemiology, Department of Public Health and Primary Care, University of Cambridge, Strangeways Research Laboratory, Cambridge, CB1 8RN UK; 14 Department of Epidemiology and Biostatistics, Case Western Reserve University, Cleveland, 44106-7219 OH USA; 15 Seidman Cancer Center, University Hospitals, Cleveland, 44106 OH USA; 16 University of Cambridge, Department of Clinical Neurosciences, Cambridge, CB2 0QQ UK; 17 Institute of Population Health, University of Manchester, Manchester, M13 9PL UK; 18 Warwick Medical School, University of Warwick, Coventry, CV4 7AL UK; 19 Division of Cancer Epidemiology and Genetics, National Cancer Institute, NIH, Bethesda, 21701 MD USA; 20 Department of Medical Epidemiology and Biostatistics, Karolinska Institute, Stockholm, SE-171 77 Sweden; 21 Epidemiology Research Program, American Cancer Society, 250 Williams Street, Atlanta, 30303 GA USA; 22 SWOG Statistical Center, Fred Hutchinson Cancer Research Center, Seattle, 98109-1024 WA USA; 23 Australian Prostate Cancer Research Centre-Qld, Institute of Health and Biomedical Innovation and School of Biomedical Science, Queensland University of Technology, Brisbane, 4059 Queensland Australia; 24 Translational Research Institute, Brisbane, 4102 Queensland Australia; 25 University College London, Department of Applied Health Research, London, WC1E 7HB UK; 26 Centre for Cancer Genetic Epidemiology, Department of Oncology, University of Cambridge, Strangeways Laboratory, Cambridge, WC1E 7HB UK; 27 Department of Medical Biochemistry and Genetics, Institute of Biomedicine, University of Turku, Turku, FI-20014 Finland; 28 Tyks Microbiology and Genetics, Department of Medical Genetics, Turku University Hospital, Hospital, 20521 Finland; 29 BioMediTech, University of Tampere, Tampere, FI-33014 Finland; 30 Division of Nutritional Epidemiology, Institute of Environmental Medicine, Karolinska Institutet, SE-171 77 Sweden; 31 Institute of Cancer Sciences, University of Manchester, Manchester Academic Health Science Centre, Radiotherapy Related Research, The Christie Hospital NHS Foundation Trust, Manchester, M13 9PL UK; 32 CeRePP, Pitie-Salpetriere Hospital, Paris, F-75020 France; 33 UPMC Univ Paris 06, GRC N°5 ONCOTYPE-URO, CeRePP, Tenon Hospital, Paris, F-75020 France; 34 Department of Molecular Medicine, Aarhus University Hospital, Aarhus, N 8200 Denmark; 35 Department of Clinical Medicine, Aarhus University, Aarhus, N 8200 Denmark; 36 Department of Medical Genetics, Oslo University Hospital, Oslo, 0424 Norway; 37 University of Cambridge, Department of Oncology, Addenbrooke’s Hospital, Cambridge, CB2 0QQ UK; 38 Cancer Research UK Cambridge Research Institute, Li Ka Shing Centre, Cambridge, CB2 0RE UK; 39 Nuffield Department of Surgical Sciences, University of Oxford, Oxford, OX1 2JD UK; 40 Faculty of Medical Science, University of Oxford, John Radcliffe Hospital, Oxford, OX1 2JD UK; 41 School of Social and Community Medicine, University of Bristol, Bristol, BS8 2PS UK; 42 Cancer Epidemiology, Nuffield Department of Population Health University of Oxford, Oxford, OX3 7LF UK; 43 Department of Surgical Oncology, Princess Margaret Cancer Centre, Toronto, M5G 2M9 Canada; 44 Department of Radiation Oncology, Icahn School of Medicine at Mount Sinai, New York, 10029 NY USA; 45 Department of Genetics and Genomic Sciences, Icahn School of Medicine at Mount Sinai, New York, 10029-5674 NY USA; 46 Centre for Molecular Oncology, Barts Cancer Institute, Queen Mary University of London, John Vane Science Centre, London, EC1M 6BQ UK; 47 Cancer Epidemiology and Intelligence Division, The Cancer Council Victoria, Melbourne, Victoria, 3004 Australia; 48 Centre for Epidemiology and Biostatistics, Melbourne School of Population and Global Health, The University of Melbourne, Melbourne, VIC 3010 Australia; 49 Division of Urologic Surgery, Brigham and Womens Hospital, Boston, 02115 MA USA; 50 Fundacion Publica Galega de Medicina Xenomica-SERGAS, Grupo de Medicina Xenomica, CIBERER, IDIS, Santiago de Compostela, 15706 Spain; 51 Centre for Research in Environmental Epidemiology (CREAL), Barcelona Institute for Global Health (ISGlobal), Barcelona, 08003 Spain; 52 CIBER Epidemiologia y Salud Publica (CIBERESP), Madrid, 28029 Spain; 53 IMIM (Hospital del Mar Research Institute), Barcelona, 08003 Spain; 54 Universitat Pompeu Fabra (UPF), Barcelona, 08002 Spain; 55 Department of Cancer Epidemiology, Moffitt Cancer Center, Tampa, 33612 USA; 56 Division of Public Health Sciences, Fred Hutchinson Cancer Research Center, Seattle, Washington 98109-1024 USA; 57 Department of Epidemiology, School of Public Health, University of Washington, Seattle, Washington 98195 USA; 58 International Hereditary Cancer Center, Department of Genetics and Pathology, Pomeranian Medical University, Szczecin, 70-115 Poland; 59 Faculty of Health and Medical Sciences, University of Copenhagen, Copenhagen, 2200 Denmark; 60 Department of Clinical Biochemistry, Herlev and Gentofte Hospital, Copenhagen University Hospital, Herlev, 2200 Denmark; 61 Division of Clinical Epidemiology and Aging Research, German Cancer Research Center (DKFZ), Heidelberg, D-69120 Germany; 62 German Cancer Consortium (DKTK), German Cancer Research Center (DKFZ), Heidelberg, D-69120 Germany; 63 Division of Preventive Oncology, German Cancer Research Center (DKFZ) and National Center for Tumor Diseases (NCT), Heidelberg, D-69120 Germany; 64 Institute for Human Genetics, University Hospital Ulm, Ulm, 89075 Germany; 65 The University of Texas M. D. Anderson Cancer Center, Department of Genitourinary Medical Oncology, Houston, 77030 TX USA; 66 Cancer Prevention Institute of California, Fremont, 94538 CA USA; 67 Department of Health Research and Policy (Epidemiology) and Stanford Cancer Institute, Stanford University School of Medicine, Stanford, 94305-5101 CA USA; 68 Department of Genetics, Portuguese Oncology Institute of Porto, Porto, 4200-072 Portugal; 69 Biomedical Sciences Institute (ICBAS), University of Porto, Porto, 4050-313 Portugal; 70 Department of Population Sciences, Beckman Research Institute of the City of Hope, Duarte, 91010 CA USA; 71 Ghent University, Faculty of Medicine and Health Sciences, Basic Medical Sciences, Gent, B-9000 Belgium; 72 Department of Surgery, Faculty of Medicine, University of Malaya, Kuala Lumpur, 50603 Malaysia; 73 Department of Urology, University of Washington, Seattle, 98195 WA USA; 74 Institute of Human Genetics, University Medical Center Hamburg-Eppendorf, Hamburg, D-20246 Germany; 75 Molecular Medicine Center, Department of Medical Chemistry and Biochemistry, Medical University, Sofia, 1431 Bulgaria; 76 Department of Oncology, Cross Cancer Institute, University of Alberta, Edmonton, AB T6G 1Z2 Alberta, Canada; 77 Division of Radiation Oncology, Cross Cancer Institute, Edmonton, AB T6G 1Z2 Alberta Canada; 78 Molecular Endocrinology Laboratory, Department of Cellular and Molecular Medicine, KU Leuven, BE-3000 Leuven, Belgium; 79 Institute of Cancer Sciences, Manchester Cancer Research Centre, University of Manchester, Manchester Academic Health Science Centre, St Mary’s Hospital, Manchester, M13 9WL UK; 80 Genomic Medicine Group, Galician Foundation of Genomic Medicine, Instituto de Investigacion Sanitaria de Santiago de Compostela (IDIS), Complejo Hospitalario Universitario de Santiago, Servicio Galego de Saude, SERGAS, Santiago De Compostela, 15706 Spain; 81 University of California San Diego, Moores Cancer Center, La Jolla, 92037 CA USA; 82 Department of Urology, Erasmus University Medical Center, Rotterdam, 3015 CE The Netherlands; 83 Cancer and Environment Group, Center for Research in Epidemiology and Population Health (CESP), INSERM, University Paris-Sud, University Paris-Saclay, Villejuif, 94807 France; 84 Clinical Gerontology Unit, University of Cambridge, Cambridge, CB2 2QQ UK; 85 Division of Genetic Epidemiology, Department of Medicine, University of Utah School of Medicine, Salt Lake City, 84112 Utah USA; 86 George E. Wahlen Department of Veterans Affairs Medical Center, Salt Lake City, 84148 UT USA; 87 The University of Surrey, Guildford, GU2 7XH Surrey UK; 88 Department of Laboratory Medicine and Pathology, Mayo Clinic, Rochester, 55905 MN USA; 89 Program in Genetic Epidemiology and Statistical Genetics, Department of Epidemiology, Harvard T.H. Chan School of Public Health, Boston, 02115 MA USA

Correction to: *Nature Communications*; 10.1038/s41467-018-06302-1; published online 4 October 2018

The original version of this Article contained an error in the spelling of a member of the PRACTICAL Consortium, Manuela Gago-Dominguez, which was incorrectly given as Manuela Gago Dominguez. This has now been corrected in both the PDF and HTML versions of the Article. Furthermore, In the original HTML version of this Article, the order of authors within the author list was incorrect. The consortium PRACTICAL consortium was incorrectly listed after Bogdan Pasaniuc and should have been listed after Kathryn L. Penney. This error has been corrected in the HTML version of the Article; the PDF version was correct at the time of publication.

