# Supplementary material for: Large-scale transcriptome-wide association study identifies new prostate cancer risk regions
Source: Nat Commun. 2018 Oct 4;9:4079. doi: 10.1038/s41467-018-06302-1 (PMC6172280; doi:10.1038/s41467-018-06302-1)
Supplement: Supplementary file 1 — Supplementary Information [file 41467_2018_6302_MOESM1_ESM.docx]

# **Large-scale transcriptome-wide association study identifies new prostate cancer risk regions**

Mancuso et al.

Supplementary Note 1

*Cross-panel and cross-gene expression model accuracy*

To determine the stability of predictive models for gene expression using cis-SNPs, we measured cross-reference panel accuracy. Due to predictive models not including the same set of SNPs resulting from QC and technological differences in the original studies, we therefore predict gene expression into the 489 samples of European ancestry from 1000 Genomes^1^ and compute Pearson correlation $R$. Specifically, we compute

$R =\rho(\mathbf{X}_{i}\mathbf{w}_{a,i}, \mathbf{X}_{i}\mathbf{w}_{b,i})$,

where $\mathbf{X}_{i}$ is the 1000Genomes genotype matrix centered around a gene $i$, $\mathbf{w}_{a,i}$ is the inferred set of eQTL weights from reference panel “a” for gene $i$ (respectively “b”), and $\rho(\cdot)$ is the sample Pearson correlation function. Using 12,701 genes with models in at least two reference panels we computed 809,513 pair-wise values of $R$ (see Supplementary Figure 5). To assess the overall similarly across tissues, we computed the average $R^{2}$ across shared genes for pairs of tissues as

$$R_{a,b}^{2} =m^{-1}{\sum_{i} \rho\left( \mathbf{X}_{i}\mathbf{w}_{a,i}, \mathbf{X}_{i}\mathbf{w}_{b,i} \right)}^{2}$$

where $m$ is the number of shared genes between reference panels a, b (see Supplementary Figure 5).

*Effect of environmental noise on TWAS*

To understand the effect of environmental noise on TWAS we performed extensive simulations starting from real genotype data in the 1000Genomes^1^ and gene boundary definitions in RefSeq^2^. We randomly sampled a gene definition using RefSeq boundaries and estimated LD (**V**) from 489 Europeans in 1000Genomes. The simulation pipeline proceeds as follows:

1. Sample genotype data for GWAS panel and eQTL independently using the MVN approximation as $\mathbf{X}_{\mathrm{GWAS}} \sim N(0, \mathbf{V})$ and $\mathbf{X}_{\mathrm{eQTL}} \sim N(0,\mathbf{V})$
2. Sample $\mathbf{y}_{\mathrm{eqtl}} = \mathbf{X}_{\mathrm{eQTL}}\boldsymbol{\beta}_{\mathrm{eQTL}} +\boldsymbol{\epsilon}_{\mathrm{eQTL}}$, where $\boldsymbol{\beta}_{\mathrm{eQTL}}$ is the causal effect vector for eQTLs and $\boldsymbol{\epsilon}$ is environmental noise. Here, a single causal eQTL is preferentially sampled near the transcription start-site and its effect is drawn such that the resulting heritability is equal to the simulation setting
3. Sample $\mathbf{y}_{\mathrm{trait}} = \left( \frac{\mathbf{X}_{\mathrm{GWAS}} \boldsymbol{\beta}_{\mathrm{eQTL}}}{\sqrt{\boldsymbol{\beta}_{\mathrm{eQTL}}^{\mathbf{T}}\mathbf{LD}\boldsymbol{\beta}_{\mathrm{eQTL}}}} \right)\alpha+\boldsymbol{\epsilon}_{\mathrm{trait}}$ where $\alpha$ is the specified effect of gene expression on trait and $\boldsymbol{\epsilon}_{\mathrm{trait}}$ is environmental noise.
4. Perform a local association scan using $\mathbf{y}_{\mathrm{trait}}$, $\mathbf{X}_{\mathrm{GWAS}}$ to get SNP summary statistics $\mathbf{z}_{\mathrm{GWAS}}$
5. Estimate expression weights $\hat{\mathbf{w}}$ using GBLUP using the eQTL samples $\mathbf{y}_{\mathrm{eqtl}}$, $\mathbf{X}_{\mathrm{eQTL}}$
6. Compute TWAS association statistic $z_{\mathrm{twas}} =\frac{{\hat{\mathbf{w}}}^{\mathbf{T}}\mathbf{z}_{\mathrm{GWAS}}}{\boldsymbol{\surd}{\hat{\mathbf{w}}}^{\mathbf{T}}\mathbf{V}\hat{\mathbf{w}}}$

We repeated these steps 250 times per 100 simulations at various values of $h_{g}^{2}$ for expression and $\alpha$. Power was computed as the proportion of times we rejected the null of no effect on trait (0.05 / 250). Overall, we find TWAS is well powered across various environmental effects on expression, and, importantly, produces unbiased results under the null (see Supplementary Figure 6).

*Comparison of expression model associations in OncoArray TWAS*

To determine the stability of predicted gene expression associations, we computed the similarity across TWAS summary statistics for predictive models fitted in the same tissue. We had three reference panels where gene expression was originally measured in blood and fitted a linear regression in R as,

$$\mathbf{z}_{a} \sim\mu\mathbf{1} + \mathbf{z}_{b}\beta$$

where $\boldsymbol{z}_{a}$ is the vector of TWAS association statistics for study a (b), and $\mu, \beta$ are the intercept and average effect for $a,b\in\{\text{YFS}, \text{NTR}, \text{GTEx.Whole\_Blood}\}$ (see Supplementary Figure 8). This model assumes independence of association statistics, which may be violated in practice; however, this reduces statistical power and does not lead to biased estimates.

*Effect of prediction accuracy on TWAS association statistics*

We next investigated if prediction accuracy has an observable effect on significant TWAS association statistics therefore inducing bias. Taking the normalized accuracy for each model ($\frac{R^{2}}{h_{g}^{2}}$), we regressed association magnitude against accuracy in two ways as,

$$\left| \mathbf{z} \right|\sim\mu\mathbf{1} + \mathbf{a}\beta$$

$$\boldsymbol{z}^{2} \sim\mu\boldsymbol{1} + \mathbf{a}\beta$$

where $\mathbf{z}$ are are the transcriptome-wide significant association statistics at tissue-specific models, $\mathbf{a}$ is the estimated $\frac{R^{2}}{h_{g}^{2}}$ at each model, and $\mu, \beta$ are the intercept and average effect. Neither approach showed evidence of an effect (see Supplementary Figure 9). We also tested for difference between average $\frac{R^{2}}{h_{g}^{2}}$ computed in transcriptome-wide significant models versus non-significant models and found a significant enrichment for overall accuracy at significant hits (0.71 vs 0.65; one-sided Mann-Whitney-U $P = 3.29 \times{10}^{-10}$). Next, we quantified if significant genes are biased towards those with models in a single tissue. Here we compared the average total number of models for genes with at least one significant association to those with none. Of the 217 significant genes, we found 2,132 / 217 = 9.8 models on average, compared with 107,038 / 16,172 = 6.6 for genes lacking an association, which suggests that significant genes tend to be identified more frequently in robustly predicted genes. Next, we repeated the analysis the focusing on the 16,389 total genes captured across models of total expression and models of alternative splicing events and found 5,022 genes exhibited heritable gene (splicing) expression levels and trained predictors in a single panel (i.e. “single-panel” genes). Of the 217 genes with at least one model reaching transcriptome-wide significance, only 33 were single-panel genes. Therefore, the odds-ratio is 0.37 (95CI = [0, 0.55]; one-sided Fisher’s exact $P=1.13 \times{10}^{-7}$), which shows that transcriptome-wide significant genes are actually depleted of single-panel genes; this suggests that genes with models in multiple panels are more likely to show as transcriptome-wide significant.

*Comparison of associations in GTEx v6p TWAS versus GTEx v7 PrediXcan*

To partially validate our results, we downloaded independently trained expression models using GTEx v7 data for S-PrediXcan^3^ (i.e. a summary-based pipeline similar to FUSION) and performed a TWAS using the OncoArray summary statistics. For the 59,861 GTEx models overlapping FUSION and S-PrediXcan we computed the Pearson correlation between TWAS association statistics and found a large degree of similarity (see Supplementary Figure 10).

*Quantifying long-range tagging of GWAS risk at novel risk regions*

To quantify the impact of long-range tagging between SNPs at known risk regions in the OncoArray PrCa GWAS and newly identified regions in using TWAS, we performed a two-step approach. We first predicted gene expression into 489 1000 Genomes samples with European ancestry^1^ using all transcriptome-wide significant models at 1Mb regions not containing a genome-wide significant SNP (see Table 1). We next computed the squared Pearson correlation between predicted expression levels and genotypes at all genome-wide significant SNPs falling on the same chromosome (see Supplementary Data 4). Overall, we observed little evidence (max $R^{2} = 0.03$) for observed TWAS signal at novel risk regions to be explained by known risk on the same chromosome.

*Bayes factors and Bayesian prioritization*

To prioritize TWAS associations we develop a Bayesian framework that adopts Bayes factors to represent the evidence of a gene being causal at a TWAS risk region. Bayes factors are defined to be the ratio between the marginal evidence for a model $M_{1}$ and a null model $M_{0}$. That is,

$$BF= \frac{\Pr\left( \mathrm{Data} \right|M_{1})}{\Pr\left( \mathrm{Data} \right|M_{0})}=\frac{\int\Pr\left( \mathrm{Data} \right|\theta_{1}, M_{1})\Pr\left( \theta_{1}|M_{1} \right)d\theta_{1}}{\int\Pr\left( \mathrm{Data} \right|\theta_{0}, M_{0})\Pr\left( \theta_{0}|M_{0} \right)d\theta_{0}} ,$$

where $\theta_{*}$ are the parameters for model $M_{*}$. The first term in the integral represents the data likelihood under a given model. The second term represents the prior distribution of the parameter. This framework has been investigated in numerous associations settings for complex traits and disease in the context of SNP associations^4-6^; however, here we adopt this methodology for associations at the level of predicted expression. Relying on the central limit theorem we use a normal likelihood of the data, and a normal prior on the effect of predicted gene expression on trait.

$$\Pr\left( \mathrm{Gene} i \right|\theta_{1}, M_{1})\Pr\left( \theta_{1}|M_{1} \right)=N(z_{TWAS,i}|\theta_{i}, 1)N(\theta_{i}|0, n\sigma^{2})$$

We can treat the effect of gene $i$ ($\theta_{i}$) as a nuisance parameter integrate out the effect resulting in

$$\Pr\left( \mathrm{Gene} i \right|M_{1})=N\left( z_{TWAS,i} | 0, 1+ n\sigma_{\alpha}^{2} \right).$$

Similarly, we use the normal distribution for the data likelihood under the null model $M_{0}$ where there is no effect

$$\Pr\left( \mathrm{Gene} i \right|M_{0})=N\left( z_{TWAS,i} | 0, 1 \right).$$

We combine these two to compute the Bayes factor at the $i$th gene as,

$$BF_{i}=\frac{N(z_{TWAS,i}|0, 1+ n\sigma_{\alpha}^{2})}{N(z_{TWAS,i}|0, 1)}=\left( 1+n\sigma_{\alpha}^{2} \right)^{-1/2}\exp\left( \frac{z_{TWAS,i}^{2}}{2}\frac{n\sigma_{\alpha}^{2}}{1+ n\sigma_{\alpha}^{2}} \right).$$

*Simulation of TWAS associations*

To ensure that credible sets computed using posterior probabilities computed using the Bayes factor approach, we ran simulations. Here we simulated TWAS association statistics directly by sampling the non-centrality parameter and estimating the covariance structure between predicted expression. Formally, at a TWAS region we have

$$\mathbf{z}_{\mathrm{TWAS}} \sim N(\mathbf{V}_{\mathbf{GE}}\boldsymbol{\lambda,}\mathbf{V}_{\mathbf{GE}}\mathbf{)}$$

where $\boldsymbol{z}_{\mathrm{TWAS}}$ are the association statistics for tissue-specific models at the region, $\mathbf{V}_{\mathbf{GE}}$ is the correlation between tissue-specific models, $\boldsymbol{\lambda}$ is the non-centrality parameter that governs association power. We sample $\boldsymbol{\lambda}_{\mathbf{i}}\boldsymbol{\sim}N(0, n\sigma^{2})$ where the $i$th gene is causal and $\boldsymbol{\lambda}_{j} =0$ for $j\neq i$. We estimated $\mathbf{V}_{\mathbf{GE}}$ using the weights provided by FUSION, and predicting expression into the 489 European samples from the 1000 Genomes project^1^. Given the sampled association statistics $\boldsymbol{z}_{\mathrm{TWAS}}$, we then compute Bayes factors for each model using the approach described above. We find that estimated credible sets are largely unbiased and capture the causal gene proportionally to the specified credible set (see Supplementary Figure 13).

*Ontology analysis of prioritized genes highlights relevant pathways*

To understand the possible biological relevance of the 109 genes prioritized by our 90% credible sets, we computed biological pathway enrichment analysis (see Methods). We identified 256 nominally significant ($P < 0.05$) GO terms in our analysis of 11,678/16,389 (93/109) genes having at least one GO category. The top category was “positive regulation of chromatin binding” ($P = 4.92 \times{10}^{-4}$; GO:0035563; see Supplementary Data 8). We observed several categories involved in apoptosis, DNA-repair, and cell-death at nominal significance (e.g., “negative regulation of extrinsic apoptotic signaling pathway”; $P = 8.83 \times{10}^{-3}$; GO:2001237). We also found several significant pathways involved in androgen production, regulation, and receptor signaling, known to be important for PrCa^8; 9^ (e.g., “androgen secretion”; $P = 7.10 \times{10}^{-3}$; GO:0035935). Taken together, these results suggest that TWAS followed by Bayesian prioritization identifies genes involved in relevant pathways for PrCa.

Supplementary Tables

**Supplementary Table 1. Tumor-prostate models of total gene expression (alternative splicing) are stable**

|  | **Total Gene Expression** | | **Alternative Splicing** | |
| --- | --- | --- | --- | --- |
|  | **R2** | **SE** | **R2** | **SE** |
| **tumor-tumor cross-validation** | 0.0642 | 0.0015 | 0.055 | 0.0007 |
| **tumor-normal prediction** | 0.062 | 0.0022 | 0.05 | 0.001 |
| **Pred R2 / CV R2** | 97% |  | 91% |  |

We predicted tumor-prostate gene expression (alternative splicing) levels using TCGA fitted models into TCGA samples with measurements of normal prostate expression (n=50). We find that out-of-sample adjusted R2 is similar to in-sample cross-validation estimates of prediction accuracy.

**Supplementary Table 2. TWAS robustly identifies novel regions in the iCOGS PrCa GWAS**

| **Gene** | **Chr** | **Tx Start** | **iCOGS Best SNP** | **iCOGS GWAS P** | **OncoArray Best SNP** | **OncoArray GWAS P** |
| --- | --- | --- | --- | --- | --- | --- |
| RPL12 | 9 | 130213329 | rs12554049 | 1.86E-04 | rs2241167 | 7.46E-08 |
| FAM154A | 9 | 18900000 | rs7465987 | 3.77E-04 | rs1048169 | 5.54E-14 |

We ran the same TWAS pipeline using a smaller PrCa GWAS (N ~ 50k) and found 2 novel regions at 1Mb. Both of these regions contained a genome-wide significant SNP in the much larger OncoArray (N ~ 140k).

**Supplementary Table 3. Previous works overlapping eQTL with PrCa GWAS risk loci**

| **Publication** | **N Genes** | **N Genes assayed in OncoArray TWAS** | **N Genes TWAS significant** | **PubMed ID** |
| --- | --- | --- | --- | --- |
| AlOlama et al. 2014 | 7 | 4 | 1 | 25217961 |
| Grisanzio et al. 2012 | 5 | 2 | 2 | 22730461 |
| Li et al. 2014 | 30 | 26 | 16 | 24907074 |
| Penney et al. 2014 | 39 | 25 | 14 | 25371445 |
| Huang et al. 2014 | 1 | 0 | 0 | 24390282 |
| Xu et al. 2014 | 1 | 1 | 1 | 24022300 |
| Thibodeau et al. 2015 | 103 | 67 | 52 | 26611117 |
| Whitington et al. 2016 | 57 | 34 | 21 | 26950096 |

N Genes is the number of genes assayed in the original work (as described by Whitington et al. 2016 and Thibodeau et al. 2015). N Genes assayed in TWAS is the number of genes overlapping our work (significant). PubMedID is the corresponding PMID for the original eQTL overlap publication.

Supplementary Figures

**Supplementary Figure 1. Genes with significantly heritable expression (alternative splicing) levels increase with reference panel sample size.** We performed a linear regression on reference panel sample size and found a significant slope ($\beta=6.9$;$95CI=[5.24, 8.66]$; $P = 1.34 \times{10}^{-10}$), which intuitively suggests that power to identify genes with heritable expression (alternative splicing) levels strongly depends on sample size.

**
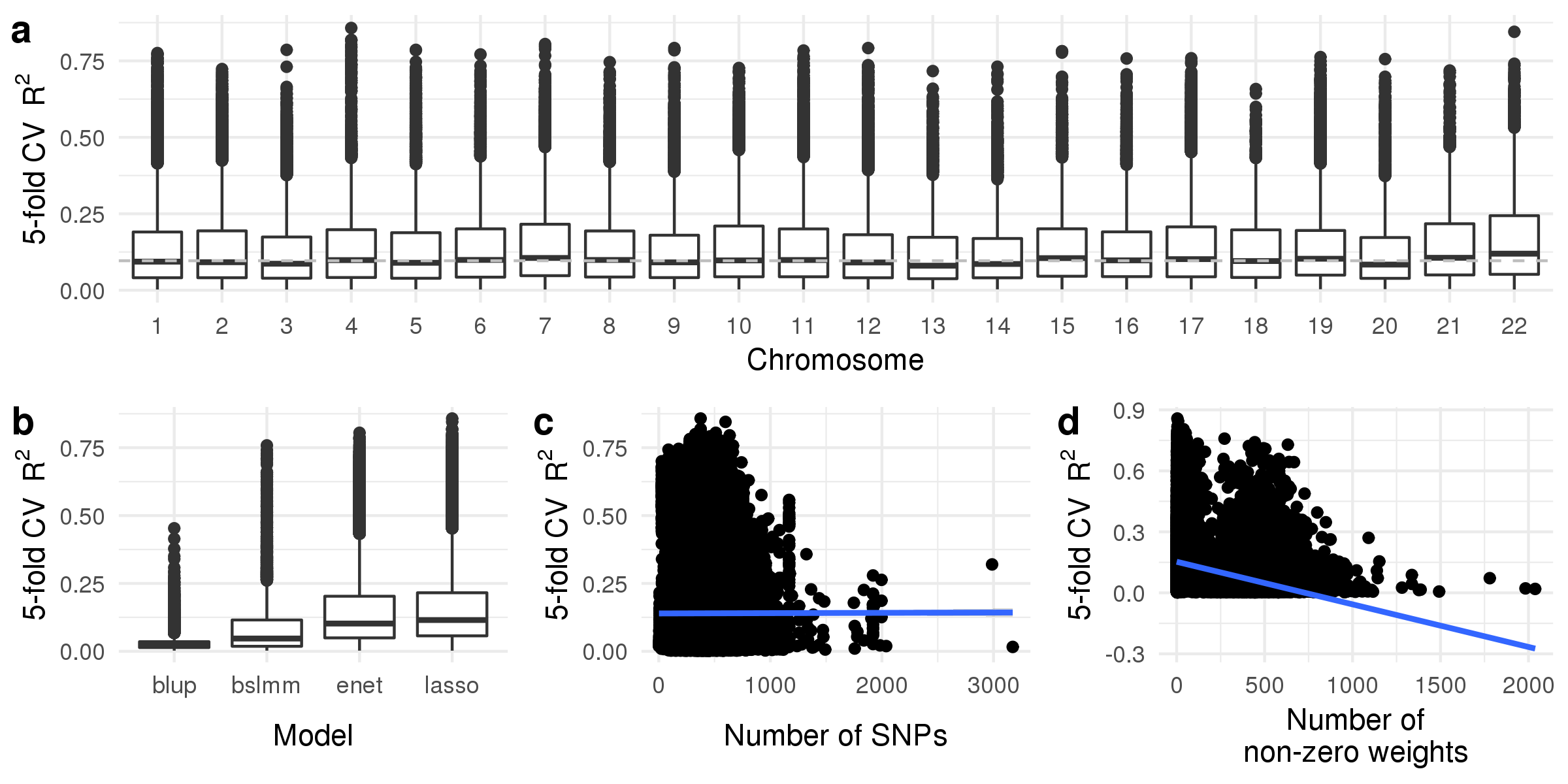
**

**Supplementary Figure 2. Model accuracy.** We estimated the average 5-fold cross-validation $R^{2}$ for each gene model stratified as a function of various model parameters. Each boxplot captures the first and third quartiles (box), median estimate (dark line), min/max (vertical lines), and outliers (points). Blue lines represent regression slopes fitted assuming a linear model. **a)** Average $R^{2}$ per chromosome. The gray dashed line represents the genome-wide median estimate of $R^{2}=0.10$. **b)** Average $R^{2}$ by inference procedure and model. **c)** Average $R^{2}$ as a function of the number of SNPs in the predictive model. Here $\beta=1.1\times{10}^{-06}$ (s.e. $=2.88\times{10}^{-6}$). **d)** Average $R^{2}$ as a function of the number of non-zero weights in the predictive model. Here $\beta=-2.1\times{10}^{-4}$ (s.e. $=2.66\times{10}^{-6}$).

**Supplementary Figure 3. Predicted expression in normal prostate replicates in out-of-sample.** We predicted normal prostate gene expression using GTEx models into TCGA samples with measured levels of normal prostate gene expression. We found the proportion of explained in-sample $R^{2}$ was highly significant $\beta= 0.41$ (s.e. = 0.04; $P = 1.7 \times{10}^{-23}$), which suggests that cis-SNP models of gene expression are stable. Here, each point represents a gene model (n=523), the blue line is a best-fit regression, and marginal histograms for in-sample cross-validated $R^{2}$ and out-of-sample $R^{2}$.

**Supplementary Figure 4. Predicted expression levels for individual genes are highly similar across panels.** We predicted expression into 489 European samples from the 1000 Genomes data and estimated the squared Pearson correlation ($R^{2}$) of predicted expression levels across individuals. We repeated this for all genes using models of both total expression and alternative splicing. The average squared Pearson correlation is $R^{2} = 0.64$.

**Supplementary Figure 5. Correlation of predicted gene expression levels between panels is highly similar.** Each entry in the lower-triangular heatmap represents the average correlation across all genes shared between tissue/expression panels. We computed averages by predicting expression into 489 European samples from the 1000 Genomes data. Next, for a gene with multiple tissue models, we estimated the Pearson correlation of predicted expression levels across samples. We repeated this over all genes and computed the mean correlation. We performed this only for models of total gene expression and did not include alternatively spliced introns.

**Supplementary Figure 6. Simulations demonstrate TWAS is well-powered and unbiased under the null.** We performed extensive simulations under the null and alternate hypothesis where gene expression impacts downstream trait. A) We observed power to be function of underlying gene expression SNP-heritability, as well as effect on downstream trait. B) Under the null (i.e. no effect on downstream trait) we observed no inflation of TWAS test statistics at any SNP-heritability strata.

**Supplementary Figure 7. Overlap with OncoArray PrCa GWAS risk regions.** A GWAS risk region (i.e. locus) is defined to be a 1Mb region containing at least one genome-wide significant SNP ($P<5 \times{10}^{-8}$). A locus is considered to overlap a gene if its index SNP (top GWAS SNP in the region) falls within 1Mb of the transcription start site. Here “eGenes” refers to any gene model put forward for association testing, and “TWAS genes” refers to genes reaching transcriptome-wide significance ($P<4.58 \times{10}^{-7}$).

**Supplementary Figure 8. Predictive models in blood are stable across datasets.** We compared TWAS association statistics using models trained in three different blood datasets (e.g., GTEx, YFS, and NTR). We observed a strong correlation for all pairs of reference datasets indicating that the latent gene expression captured by models of cis-SNPs is stable.

**Supplementary Figure 9. Prediction accuracy does not bias association at transcriptome-wide significant genes.** We regress prediction accuracy against the absolute and squared TWAS association signal and observed little evidence of bias as indicated by an estimated slope overlapping zero. A) $\beta= -0.5$; 95CI = [-0.96,-0.05]; $P = 0.03$ B) $\beta= -7.5$; 95CI = [-16.7, 1.66]; $P = 0.11$.


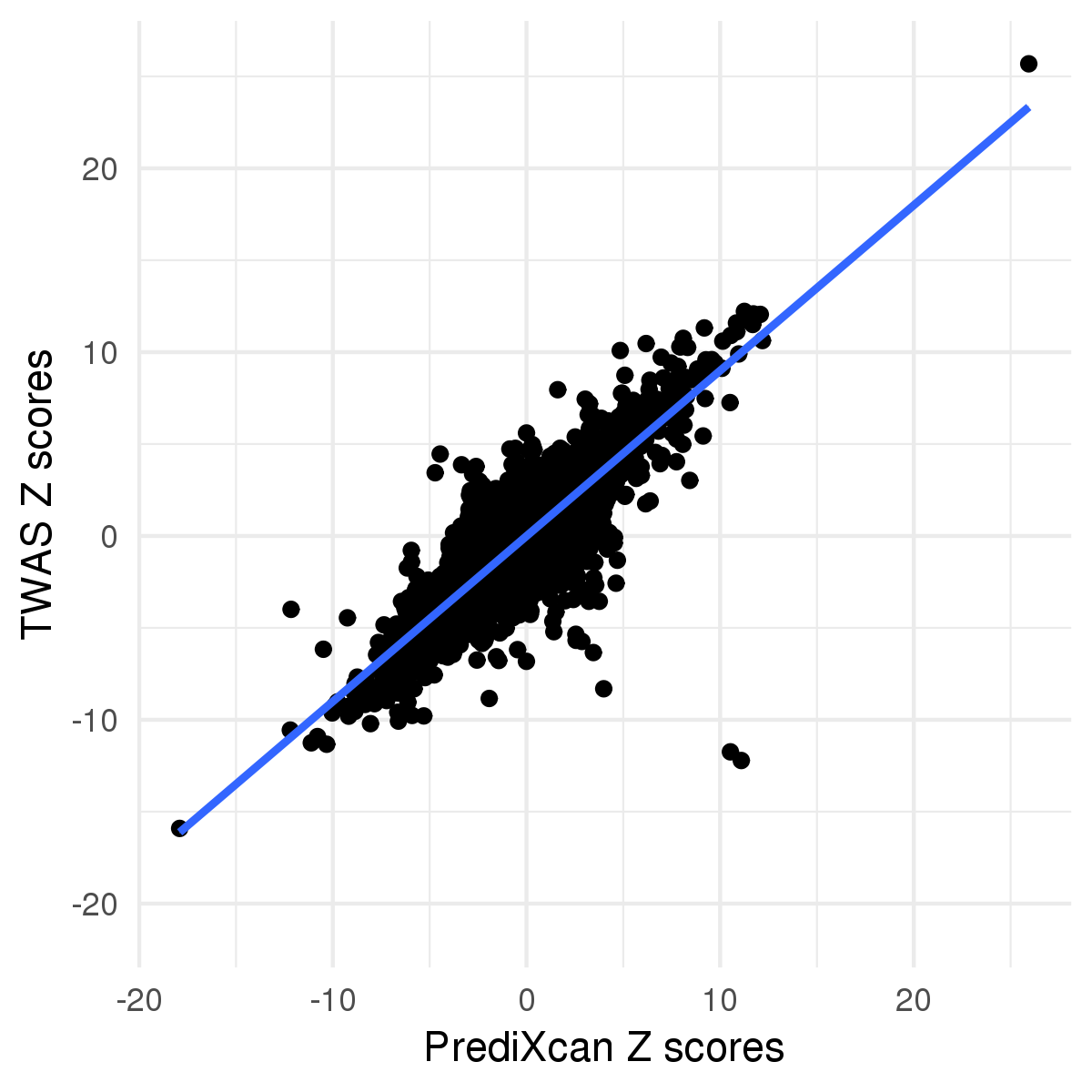


**Supplementary Figure 10. TWAS (FUSION) and PrediXcan compute similar association statistics in OncoArray with independently trained models.** We downloaded S-PrediXcan and preditive models of gene expression for GTEx v7 designed as input to S-PrediXcan. S-PrediXcan is an independent method designed to perform a summary-based TWAS. We found results on the OncoArray PrCa GWAS for the $N=56,696$ overlapping tissue-specific gene models were highly consistent with those using predictive models of GTEx v6p trained with FUSION ($\beta=0.90$; $P < 2\times{10}^{-16}$), further supporting the stability of predicting gene expression using cis-SNPs.

**Supplementary Figure 11. SNPs at novel PrCa regions have increased association signal compared to genetic background SNPs.** **A)** QQ-plot of SNP-PrCa association signal restricted to 1Mb regions with a transcriptome-wide significant gene model, but no genome-wide significant SNP. The red line indicates the identity line between observed and expected statistics. **B)** Histogram of 50 $\lambda_{GC}$ estimates in OncoArray GWAS for 4,534 SNPs randomly sampled after matching LD and MAF patterns observed in novel PrCa regions. The red line indicates the estimated $\lambda_{GC}$ value from the 4,534 SNPs in novel regions. An empirical P-value of observing $\lambda_{GC}$ estimated at novel regions compared to genome-wide background is $P=0.02$, which we computed as $P = \frac{\#\left\{ \left| \lambda_{GC,i} \right|\geq\left| \lambda_{GC} \right| \right\} + 1}{51}$.

**Supplementary Figure 12. Average TWAS association statistics for genes predicted in each expression panel.** Each bar plot corresponds to the average TWAS association statistic using only transcriptome-wide significant genes predicted in each expression panel. Lines represent 1 standard-deviation estimated using the median absolute deviation under normality assumptions. Normal and tumor prostate tissues are marked in green.

**Supplementary Figure 13. Bayesian credible sets of genes are unbiased in capturing causal genes in simulations.** We simulated TWAS statistics under a model with a single causal gene and correlations between predicted levels of expression. We then computed credible sets at various values of $\rho$. The blue line represents the fitted line for the $\rho$ threshold and the proportion of true causal genes captured genome-wide. The shaded region around the line represents the 95% confidence interval. A) Simulations with prior effect-size variance at $n\sigma^{2} = 13$. B) Simulations with prior effect-size variance at $n\sigma^{2} = 2$.

### References

1. The Genomes Project, C. (2015). A global reference for human genetic variation. Nature 526, 68-74.

2. O'Leary, N.A., Wright, M.W., Brister, J.R., Ciufo, S., Haddad, D., McVeigh, R., Rajput, B., Robbertse, B., Smith-White, B., Ako-Adjei, D., et al. (2016). Reference sequence (RefSeq) database at NCBI: current status, taxonomic expansion, and functional annotation. Nucleic Acids Research 44, D733-D745.

3. Barbeira, A.N., Dickinson, S.P., Torres, J.M., Bonazzola, R., Zheng, J., Torstenson, E.S., Wheeler, H.E., Shah, K.P., Edwards, T., Garcia, T., et al. (2017). Exploring the phenotypic consequences of tissue specific gene expression variation inferred from GWAS summary statistics. bioRxiv.

4. Chen, W., Larrabee, B.R., Ovsyannikova, I.G., Kennedy, R.B., Haralambieva, I.H., Poland, G.A., and Schaid, D.J. (2015). Fine Mapping Causal Variants with an Approximate Bayesian Method Using Marginal Test Statistics. Genetics 200, 719.

5. Wakefield, J. (2009). Bayes factors for genome-wide association studies: comparison with P-values. Genetic Epidemiology 33, 79-86.

6. Wakefield, J. (2007). A Bayesian Measure of the Probability of False Discovery in Genetic Epidemiology Studies. The American Journal of Human Genetics 81, 208-227.

7. Wang, B., Xiao, Y., Ding, B.-B., Zhang, N., Yuan, X.-b., Gui, L., Qian, K.-X., Duan, S., Chen, Z., Rao, Y., et al. Induction of tumor angiogenesis by Slit-Robo signaling and inhibition of cancer growth by blocking Robo activity. Cancer Cell 4, 19-29.

8. Hazelett, D.J., Rhie, S.K., Gaddis, M., Yan, C., Lakeland, D.L., Coetzee, S.G., Henderson, B.E., Noushmehr, H., Cozen, W., Kote-Jarai, Z., et al. (2014). Comprehensive Functional Annotation of 77 Prostate Cancer Risk Loci. PLoS Genet 10, e1004102.

9. Gusev, A., Shi, H., Kichaev, G., Pomerantz, M., Li, F., Long, H.W., Ingles, S.A., Kittles, R.A., Strom, S.S., Rybicki, B.A., et al. (2016). Atlas of prostate cancer heritability in European and African-American men pinpoints tissue-specific regulation. 7, 10979.
