# Supplementary material for: Large-scale transcriptome-wide association study identifies new prostate cancer risk regions
Source: Nat Commun. 2018 Oct 4;9:4079. doi: 10.1038/s41467-018-06302-1 (PMC6172280; doi:10.1038/s41467-018-06302-1)
Supplement: Supplementary file 2 — Description of Additional Supplementary Files [file 41467_2018_6302_MOESM2_ESM.pdf]

Description of Additional Supplementary Files

Supplementary Data 1
Description: Expression data sets used to train predictive models of gene expression. The number of gene models retained after pruning genes with non-significant cis-SNP heritability levels ($P > 0.05$) and non-significant cross-validation ($P > 0.05$). Sample size indicates the number of individuals with measured expression levels and genotype information. GTEx v6 expression panels collectively account for 449 samples. Alternative splicing events from the same gene count represent the same gene in counting the number of unique associations.

Supplementary Data 2
Description: Transcriptome-wide significant associations. Study denotes the original expression panel used to fit weights. Expression h2g captures only the cis-regulated component. Z scores represent the association strength for GWAS SNPs and TWAS genes. P-value for TWAS computed under the null of no association strength between expression and PrCa under a Normal(0, 1) distribution. An asterisk (*) indicates associations that are significant under an adaptive permutation test after accounting for 217 genes ($P < 0.05 / 217$).

Supplementary Data 3
Description: Novel risk regions in the OncoArray identified by TWAS. We assessed novelty of TWAS associations as those that did not overlap a genome-wide significant SNP within a specified window flanking the transcription-start site for a gene model.

Supplementary Data 4
Description: Predicted expression at novel regions do not tag GWAS risk SNPs. We performed a two-step procedure to identify long-range tagging between predicted gene expression at novel risk regions and known risk regions in the OncoArray. We first predicted gene (splice) expression levels at novel regions into 1000Genomes samples. We then computed the squared Pearson correlation with genotypes for all OncoArray PrCa risk SNPs on the same chromosome.

Supplementary Data 5
Description: Comparison of previous eQTL/GWAS overlap with TWAS. Tissue sample is the training expression panel used to fit gene weights to include in TWAS. TWAS.P is the association P-value for the TWAS test. An asterisk (*) in TWAS.SIG denotes if TWAS.P < 0.05 / 109170.

Supplementary Data 6
Description: 90% credible sets for TWAS regions in the OncoArray data. Block number is an identifier for each 1MB group considered for analysis, TWAS.Z is the TWAS association statistic, BP is the computed Bayes Factor for that gene model, and PIP is the posterior inclusion probability of a tissue-specific gene to be causal.

Supplementary Data 7
Description: 90% credible sets for TWAS regions in the OncoArray data collapsed to unique genes. Block number is an identifier for each 1MB group considered for analysis, TWAS.Z is the TWAS association statistic, BP is the computed Bayes Factor for that gene model, and PIP is the posterior inclusion probability of a tissue-specific gene to be causal.

Supplementary Data 8
Description: Biological pathways using credible set genes*.* GO categories refer to gene ontology category number. P-values are computed using the Wallenius approximation to the hypergeometric distribution. We list all results that are nominally significant ($P < 0.05$).
